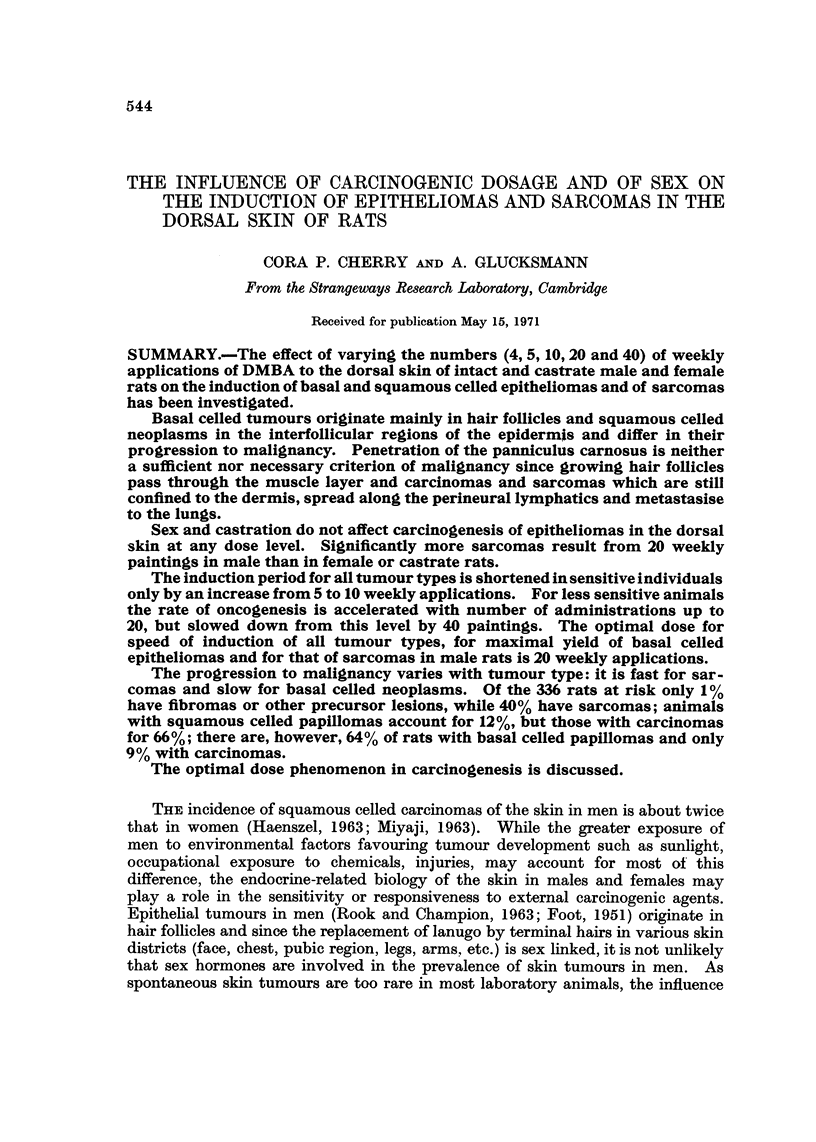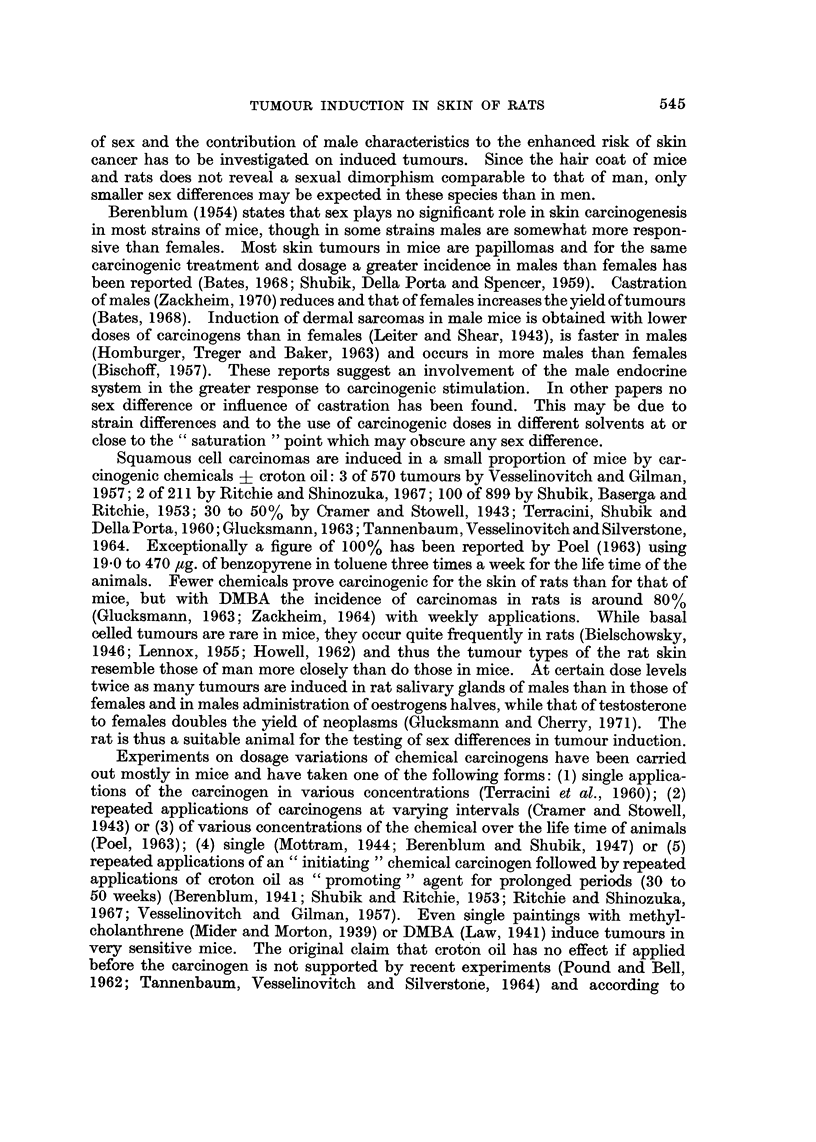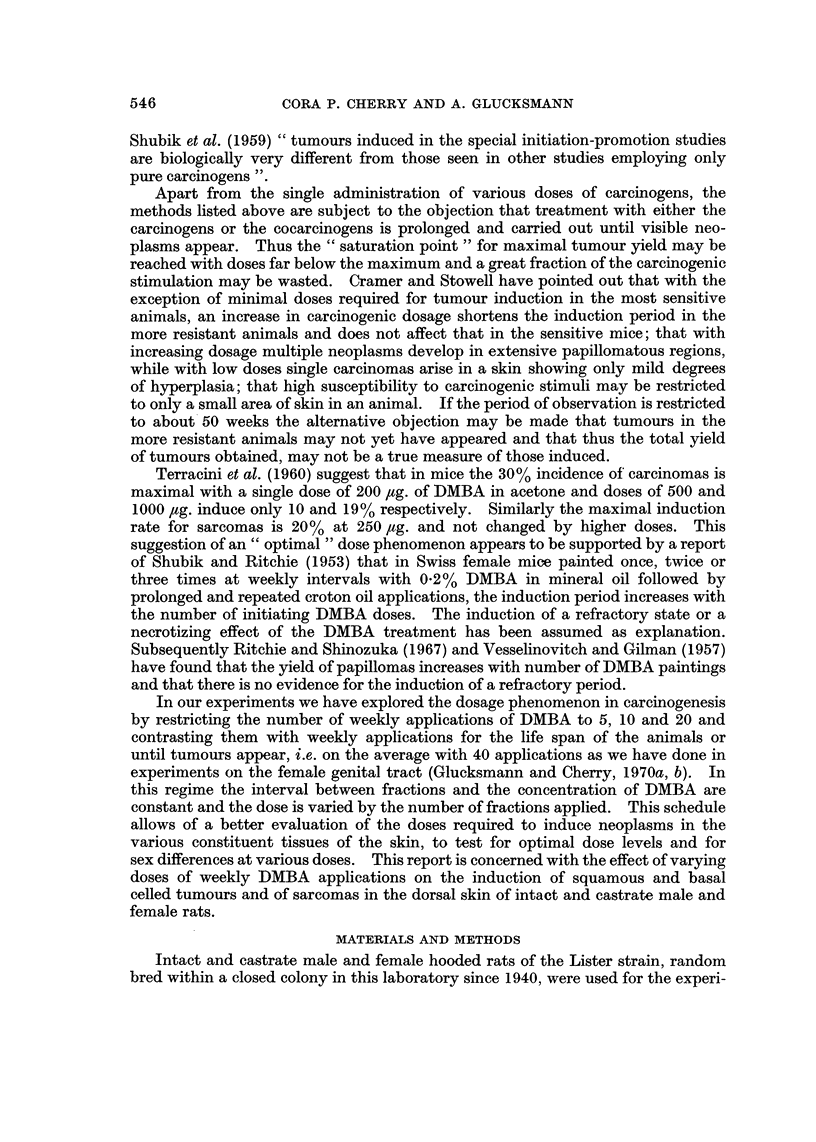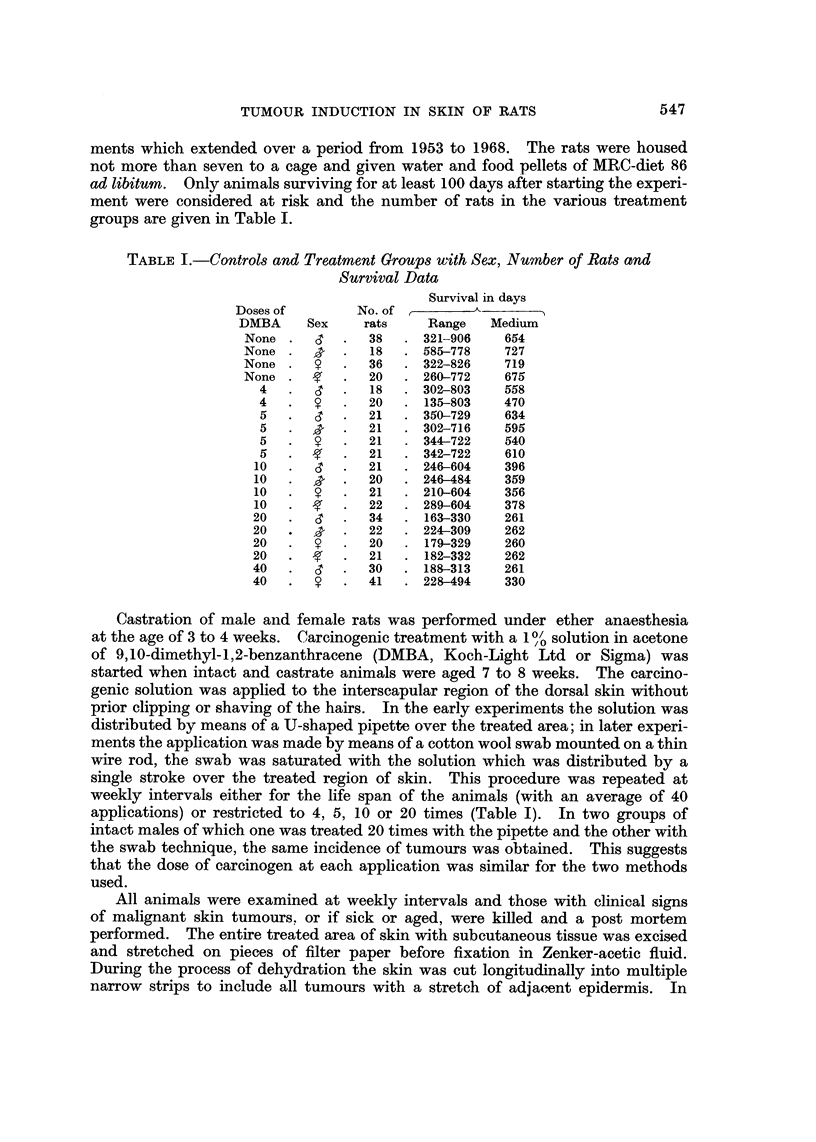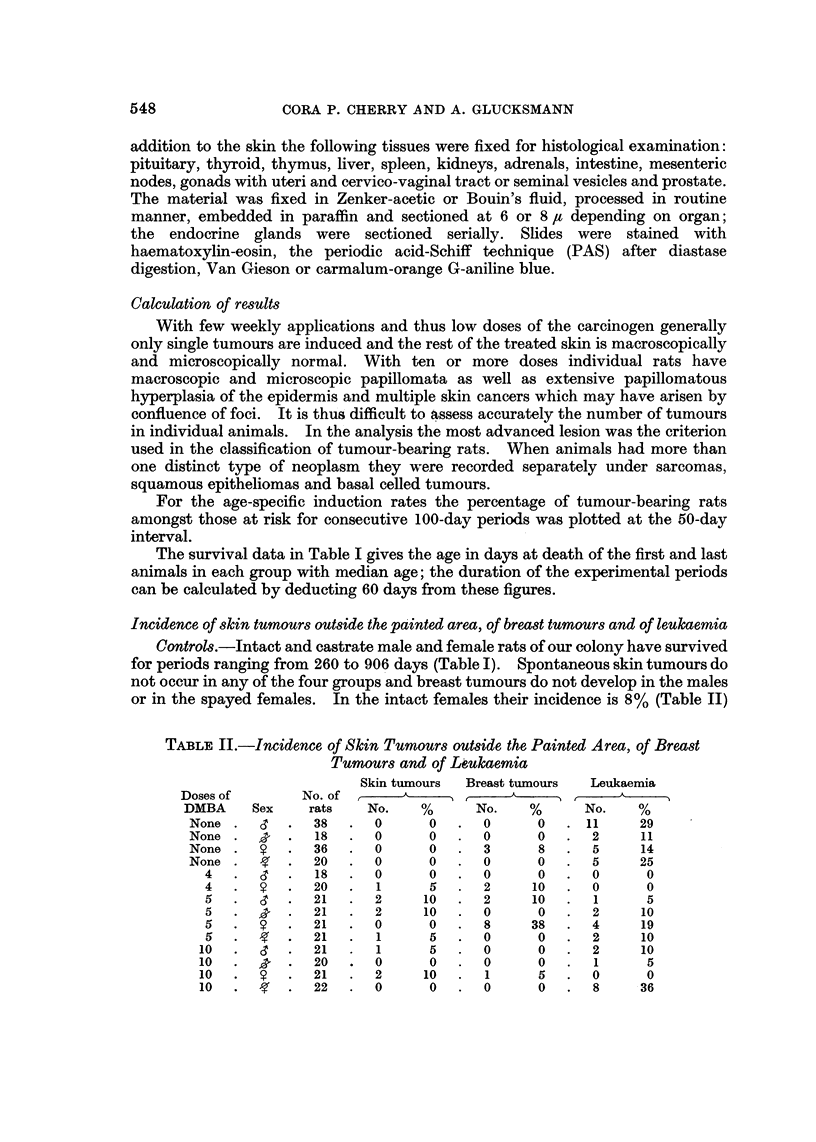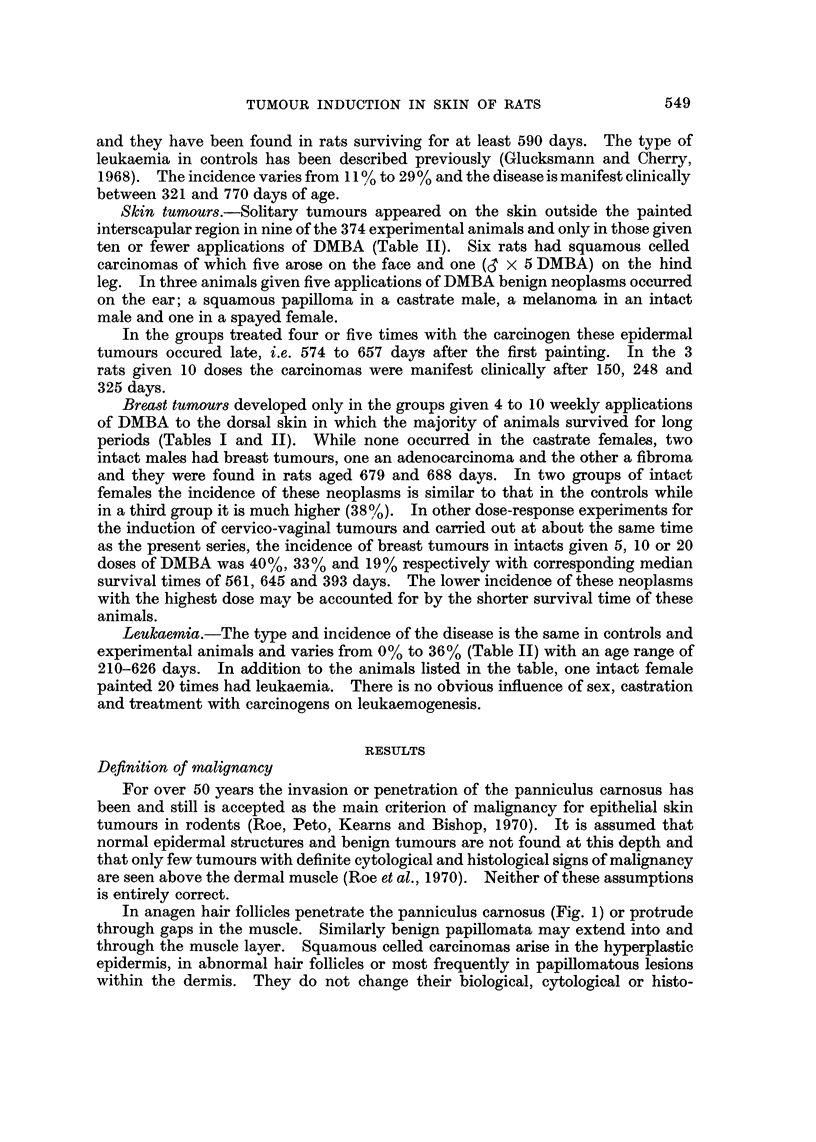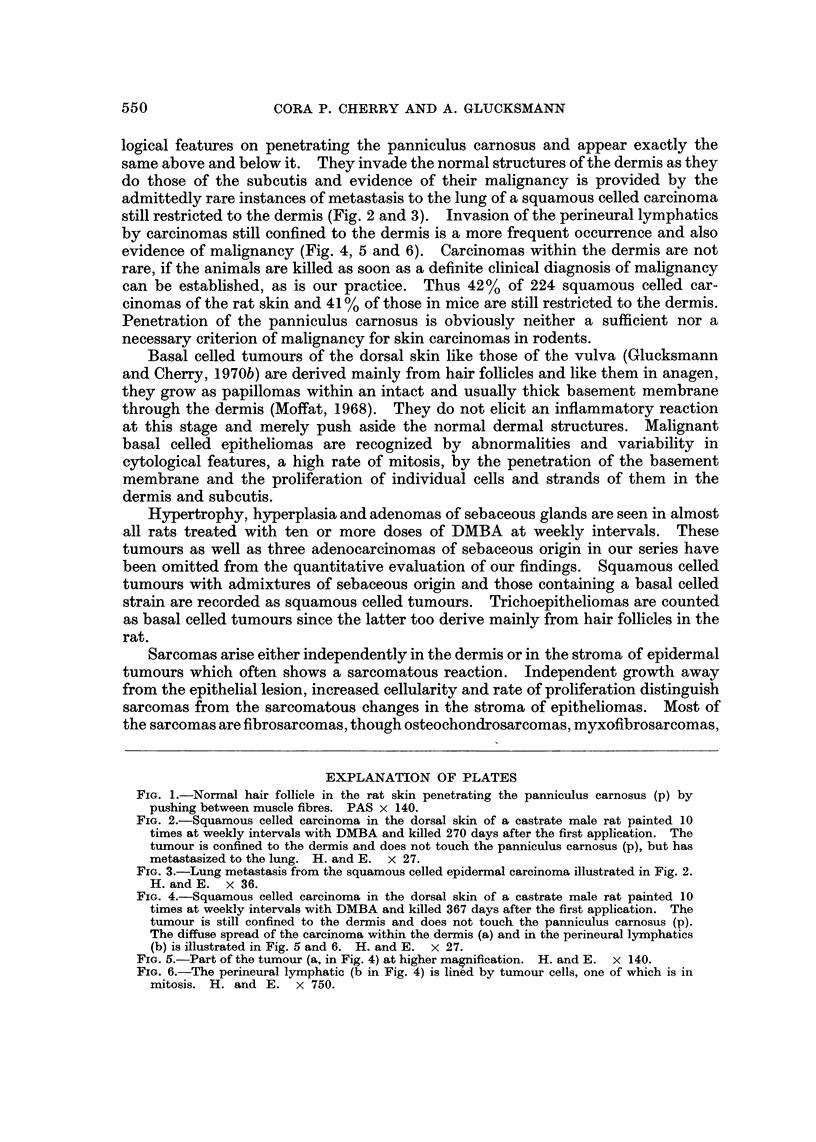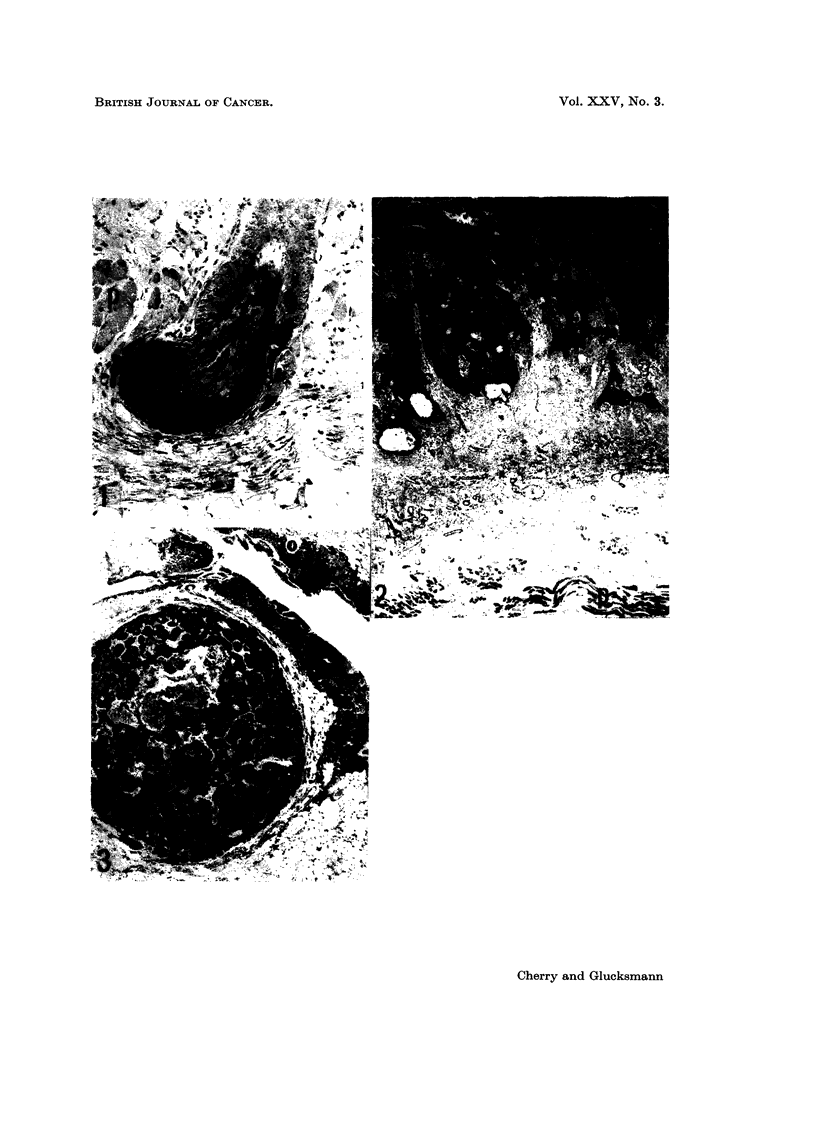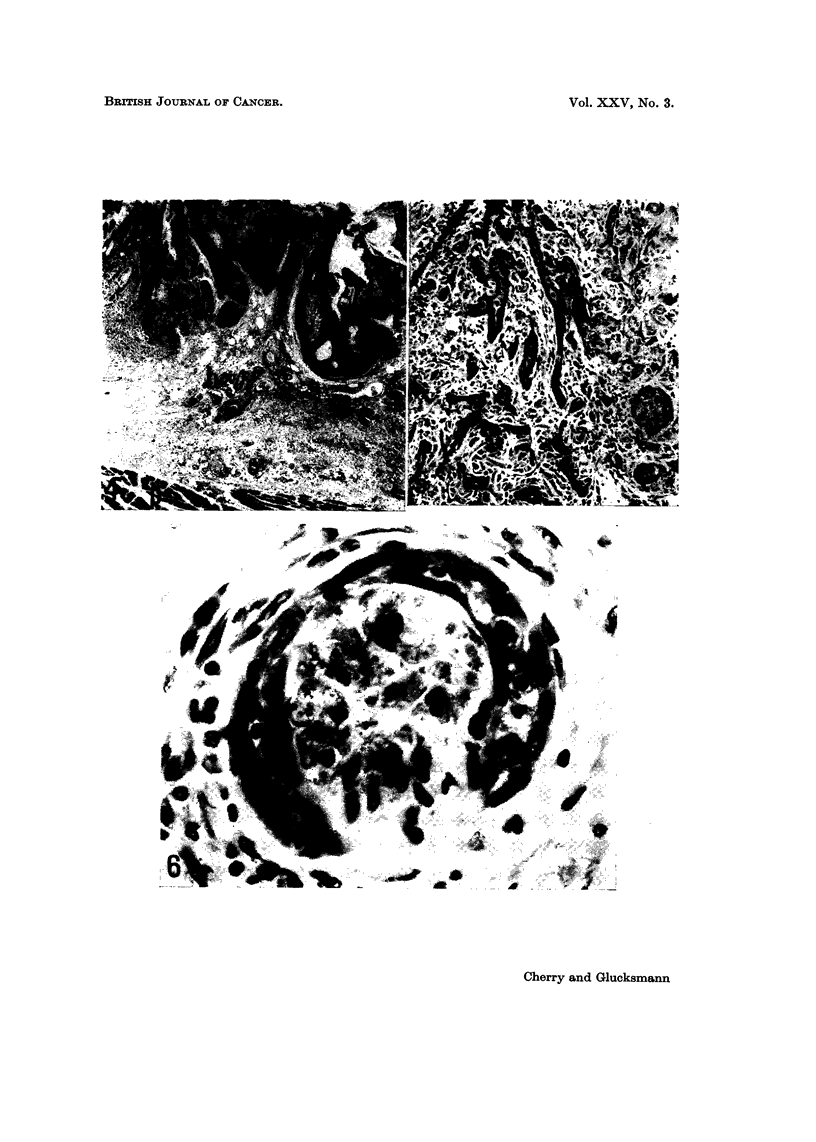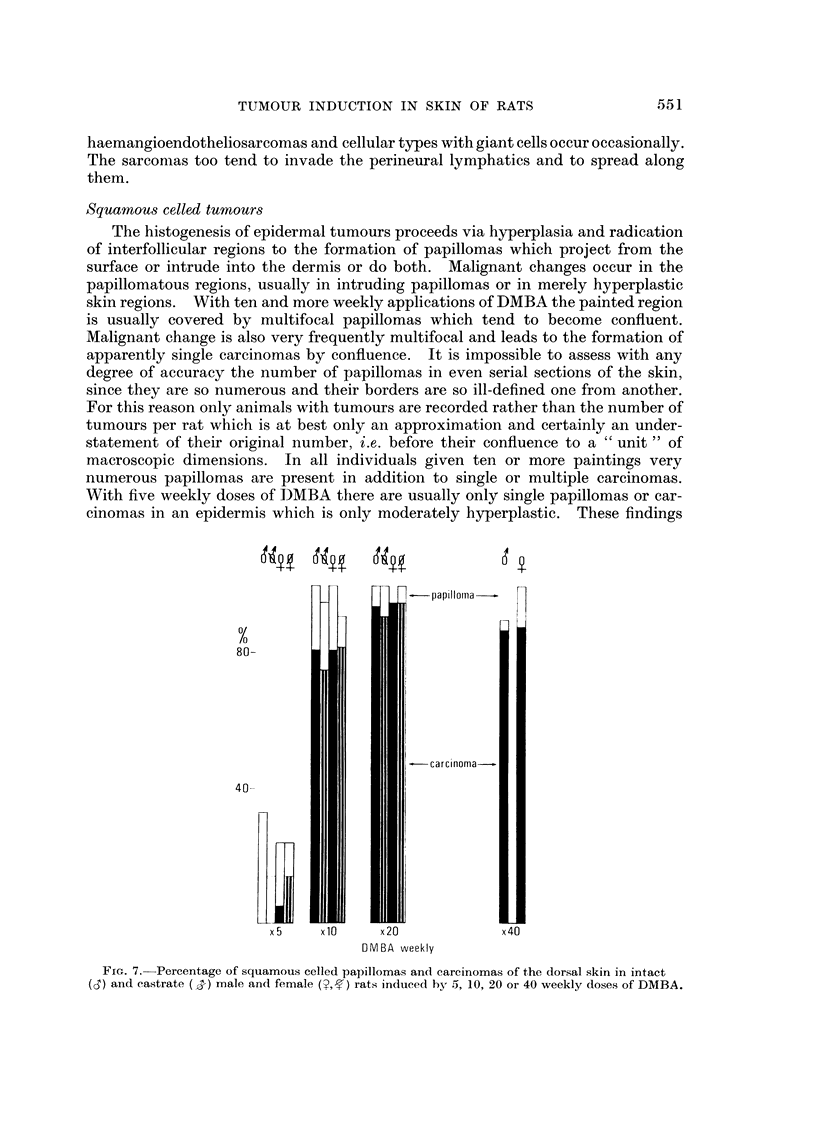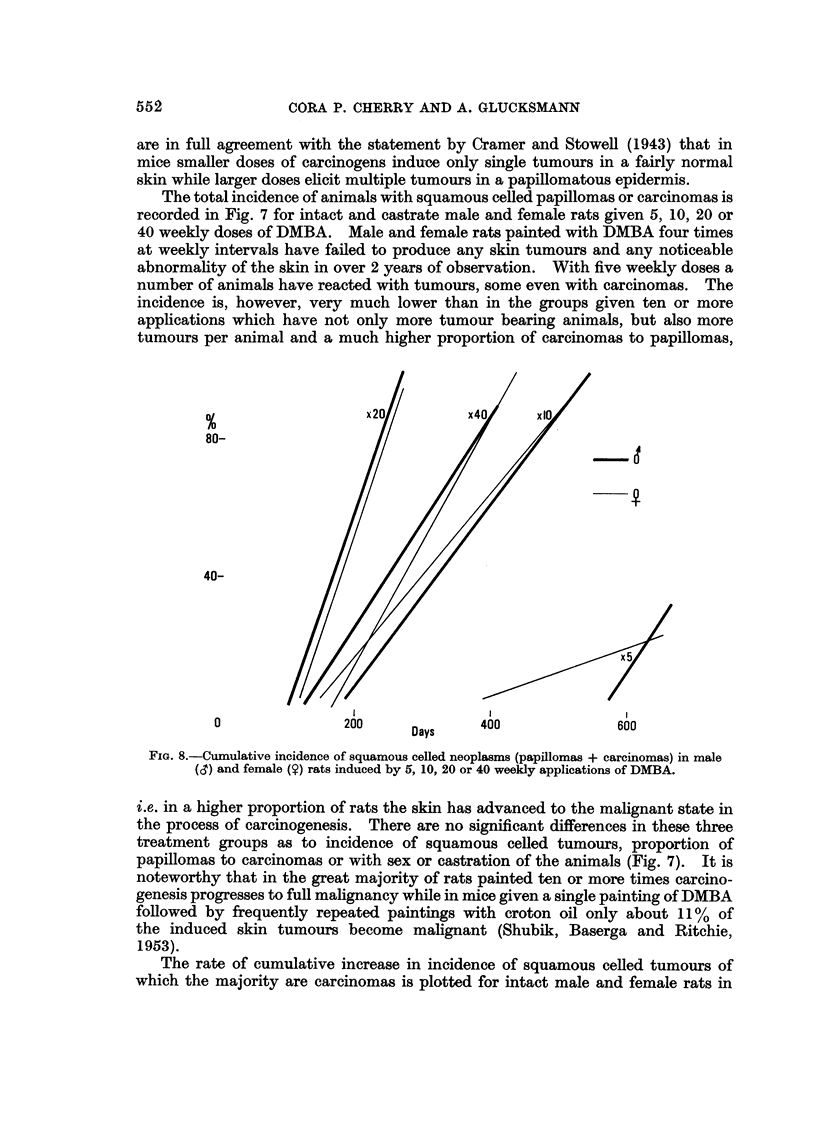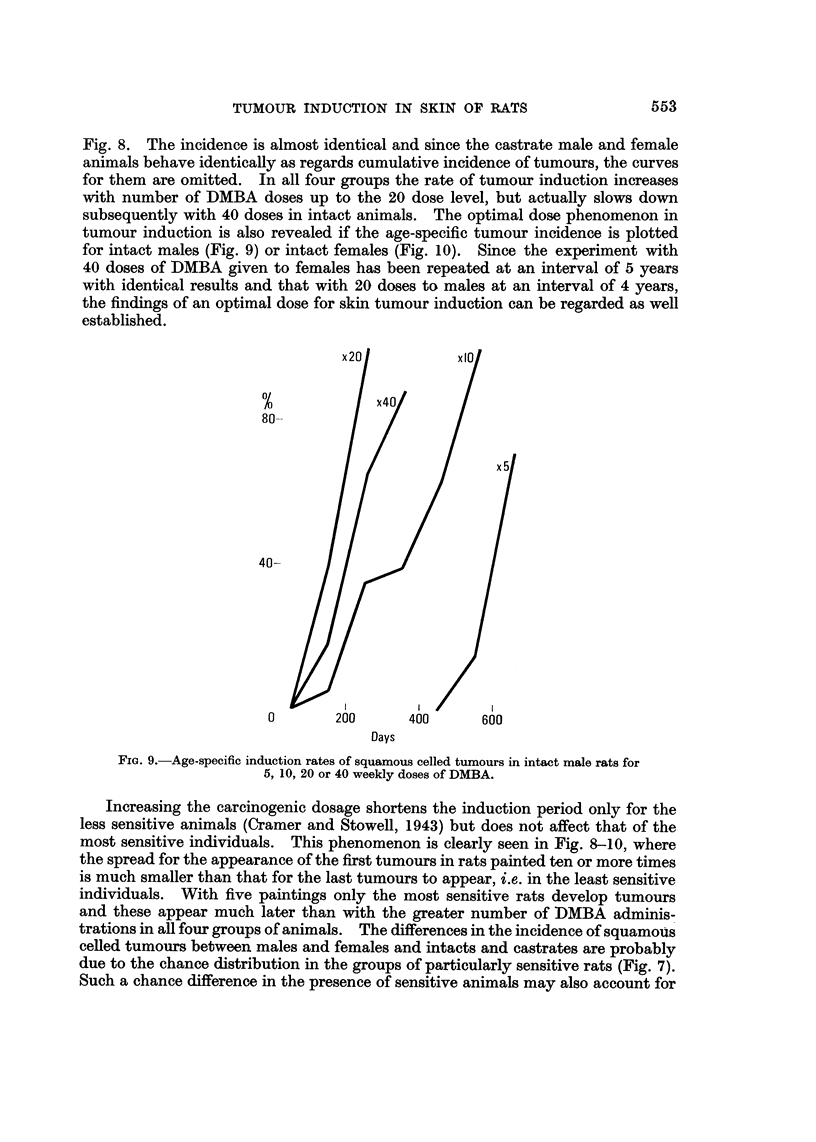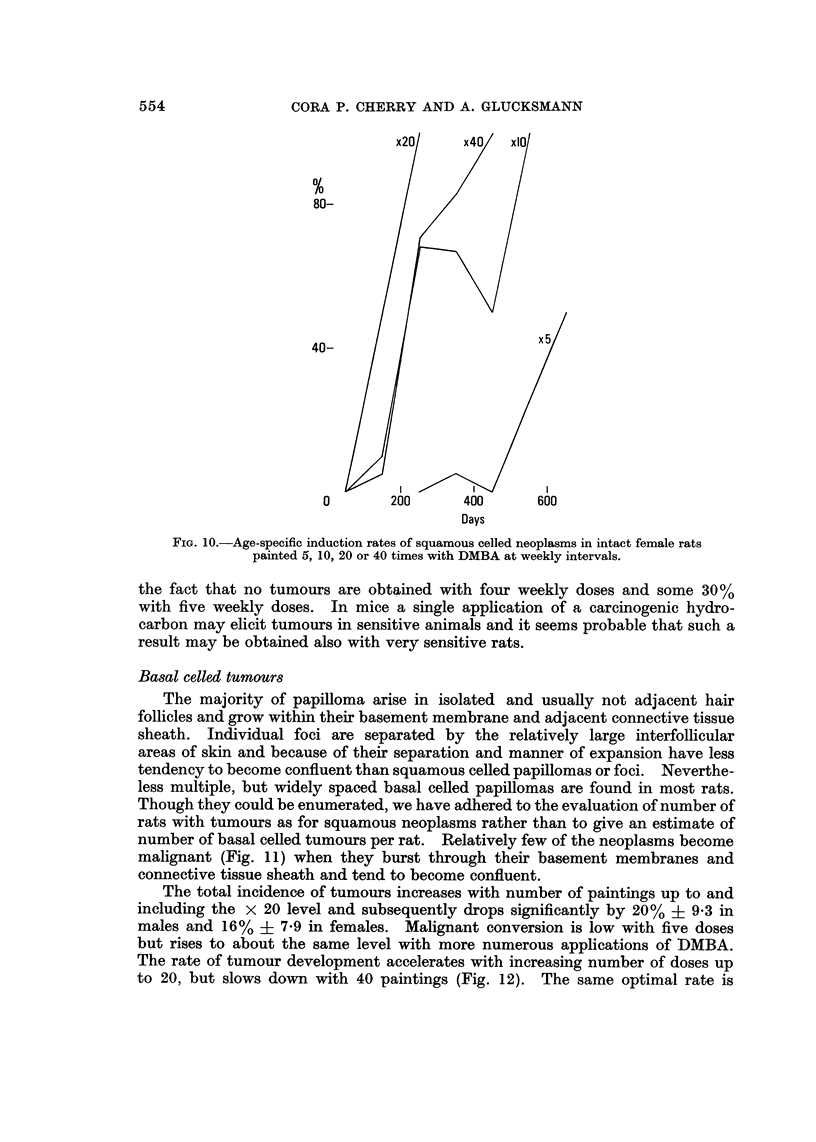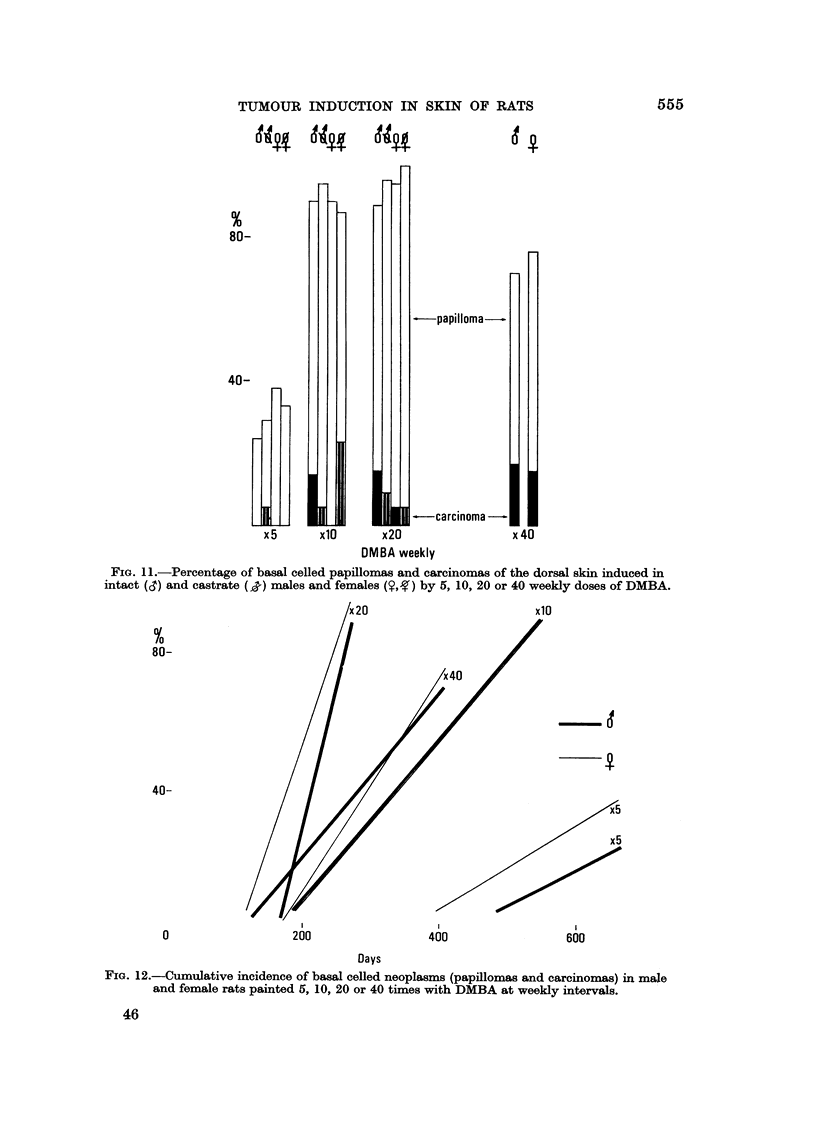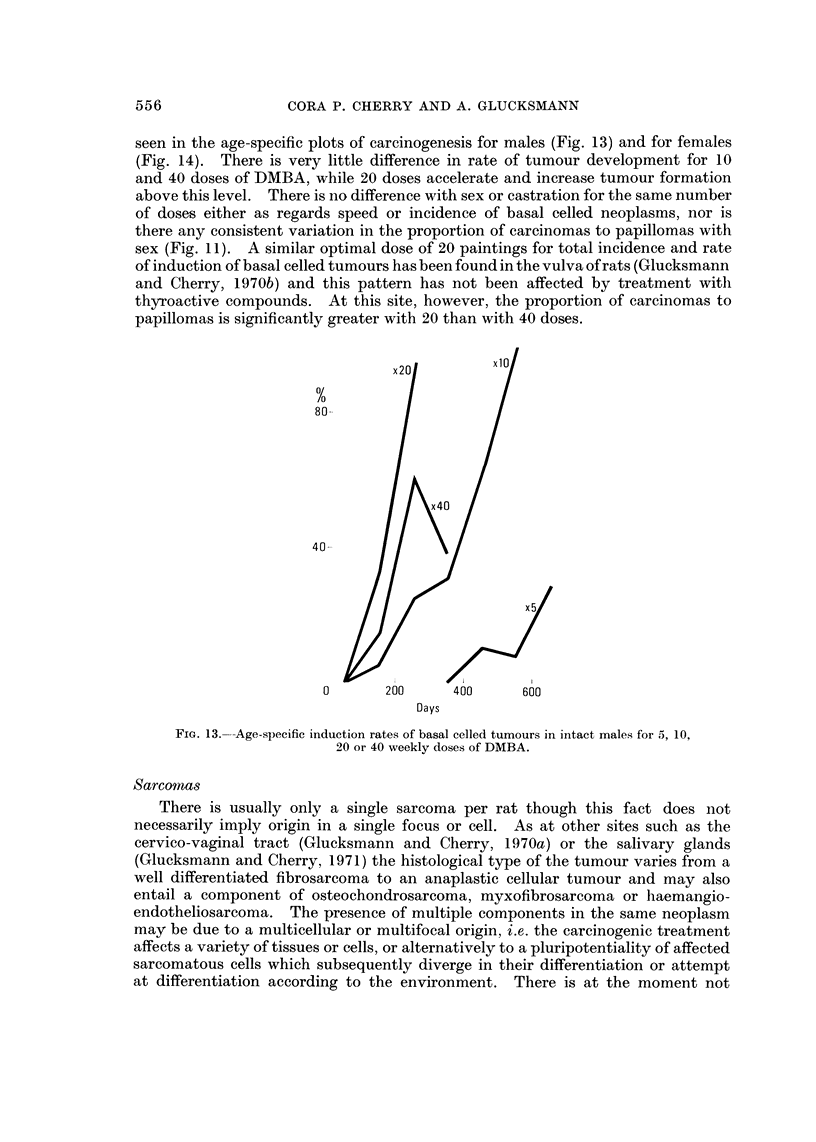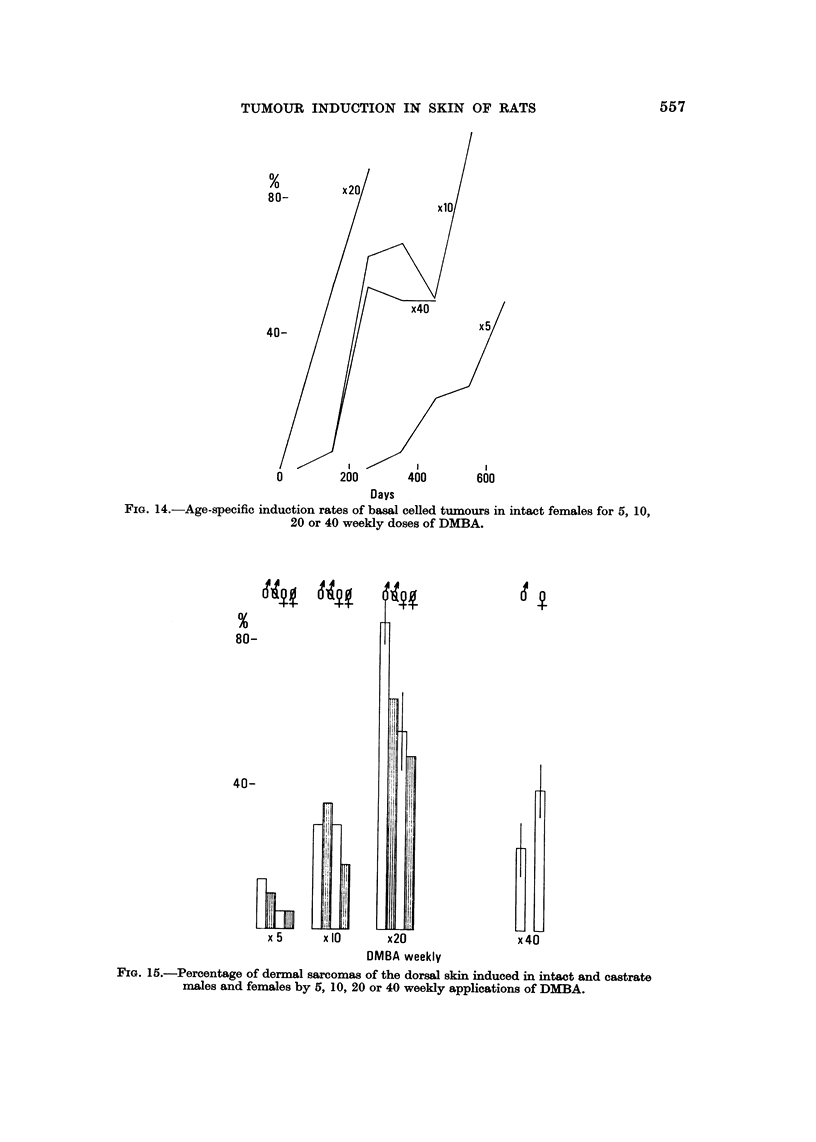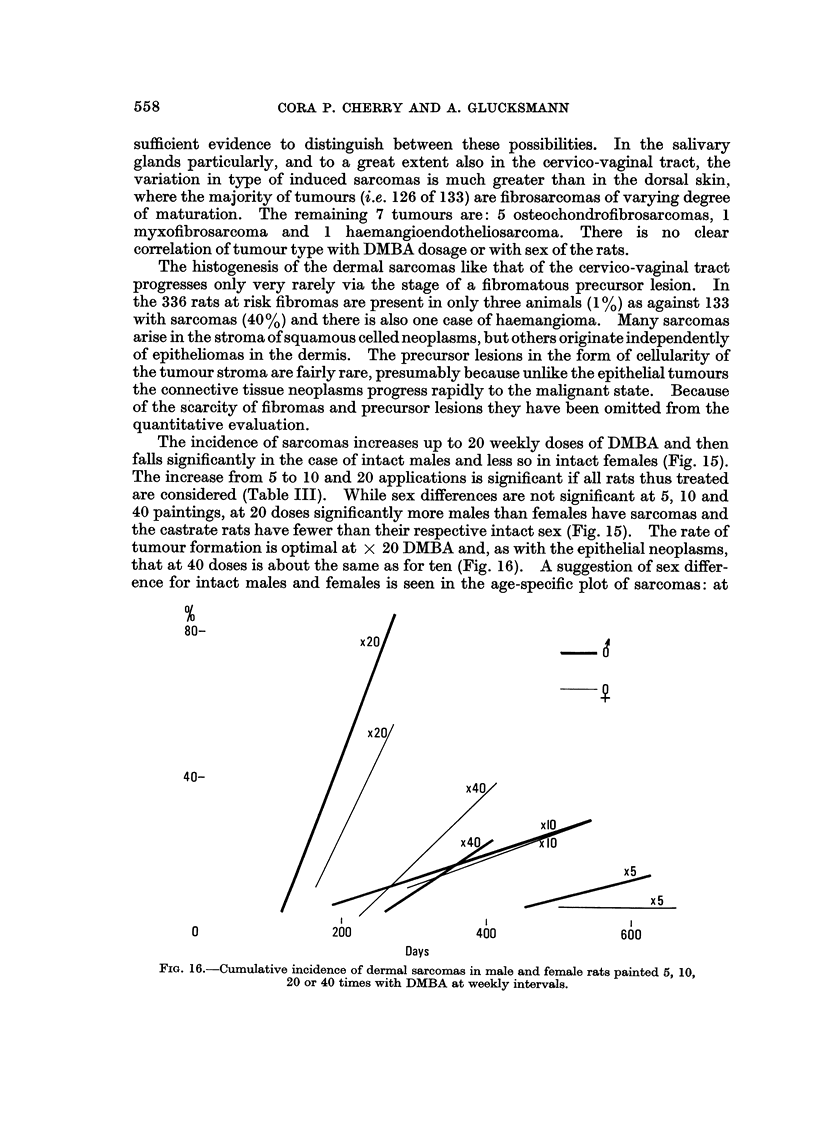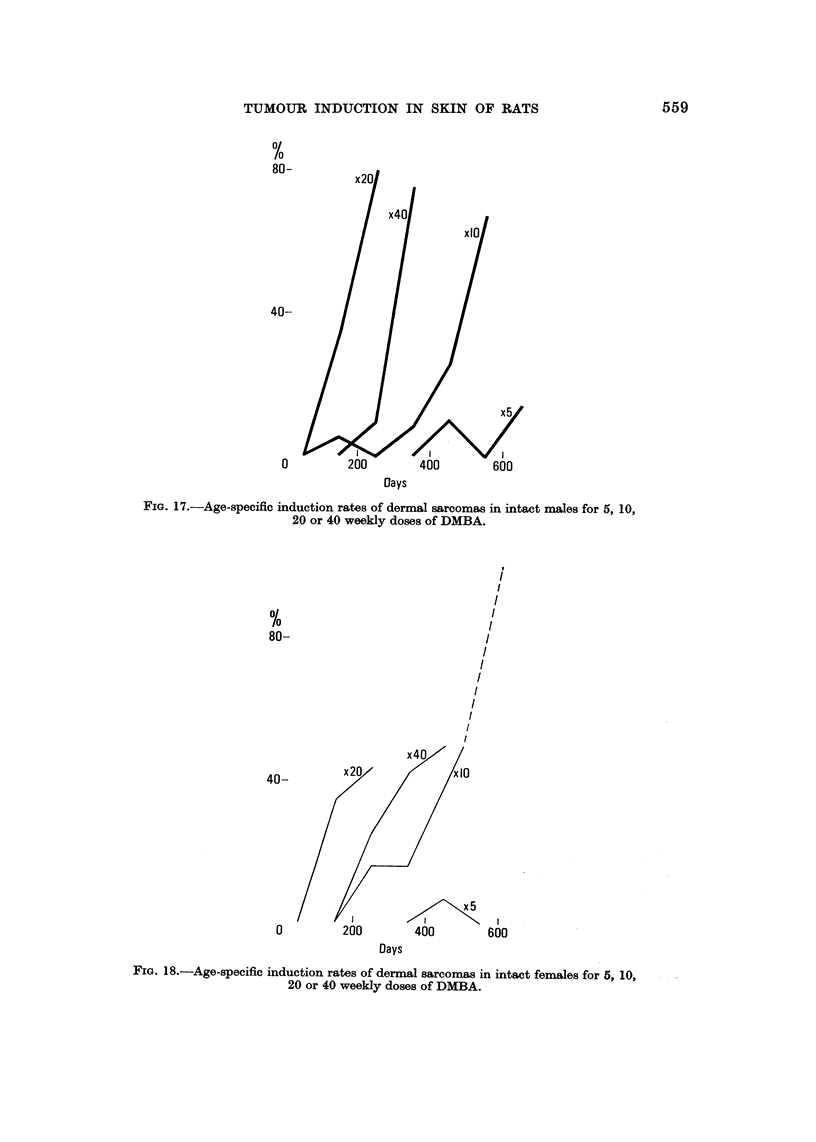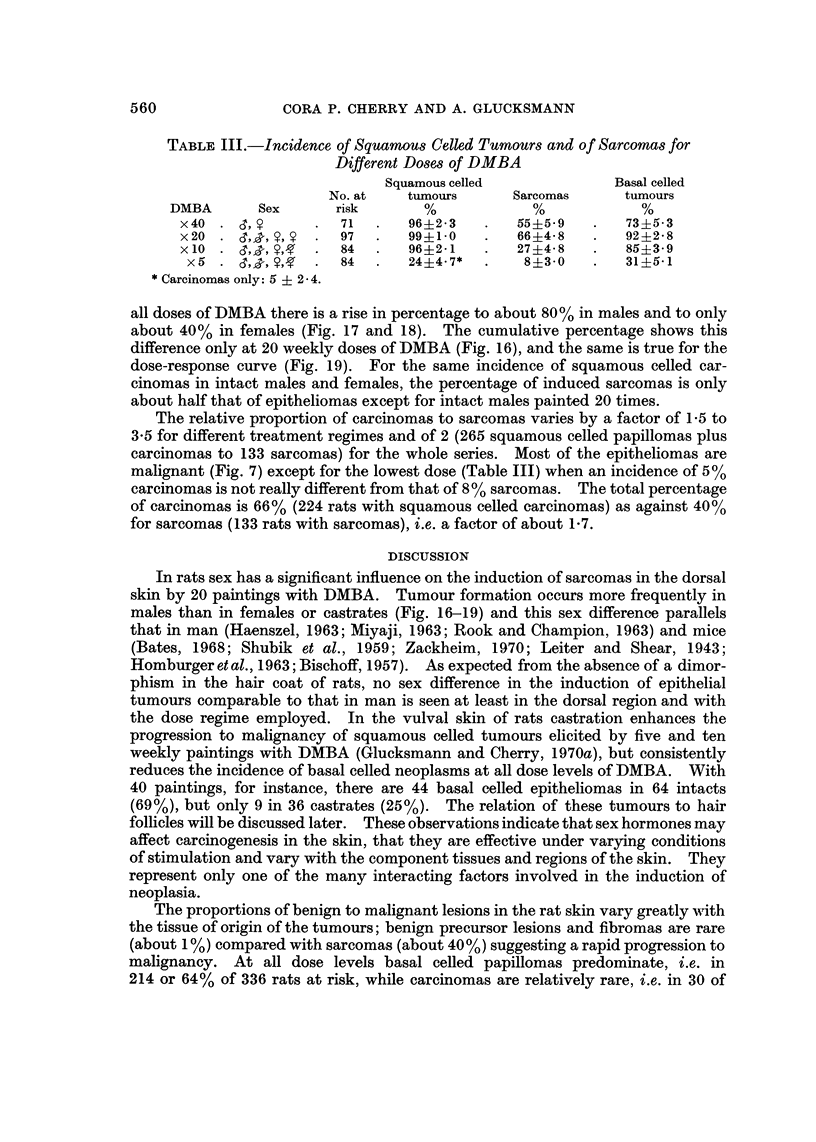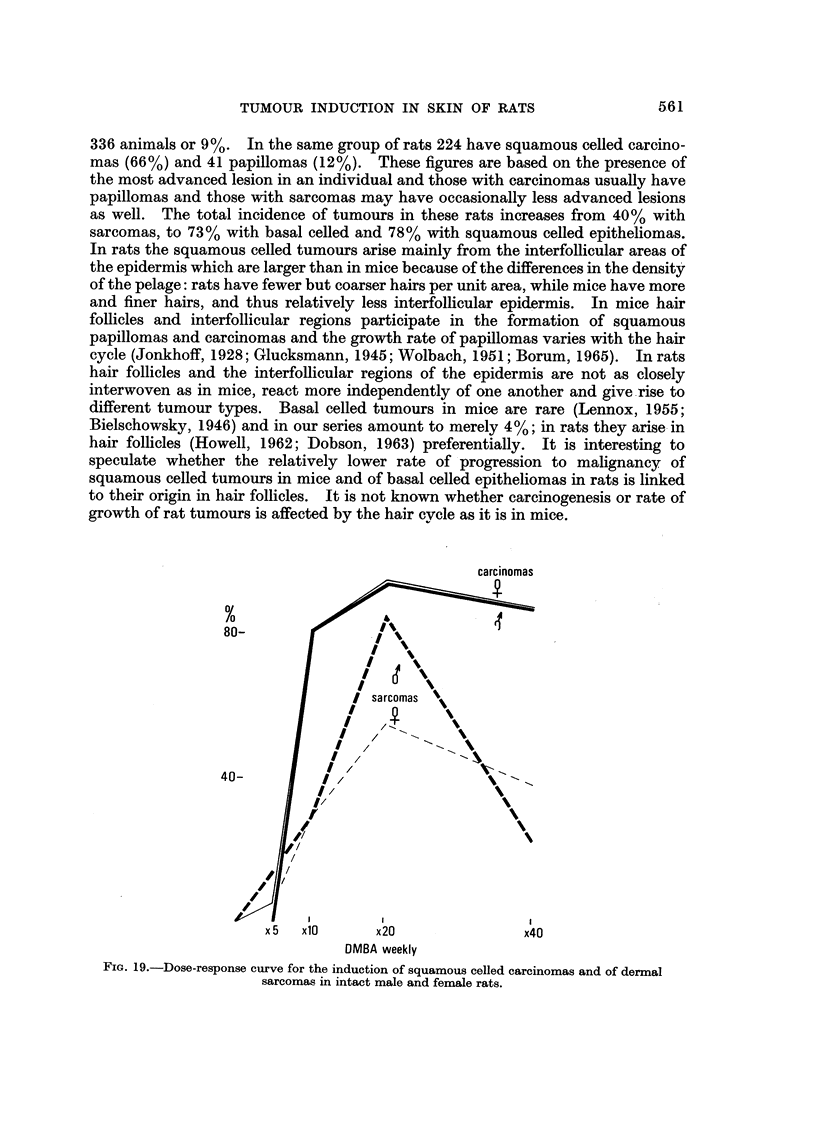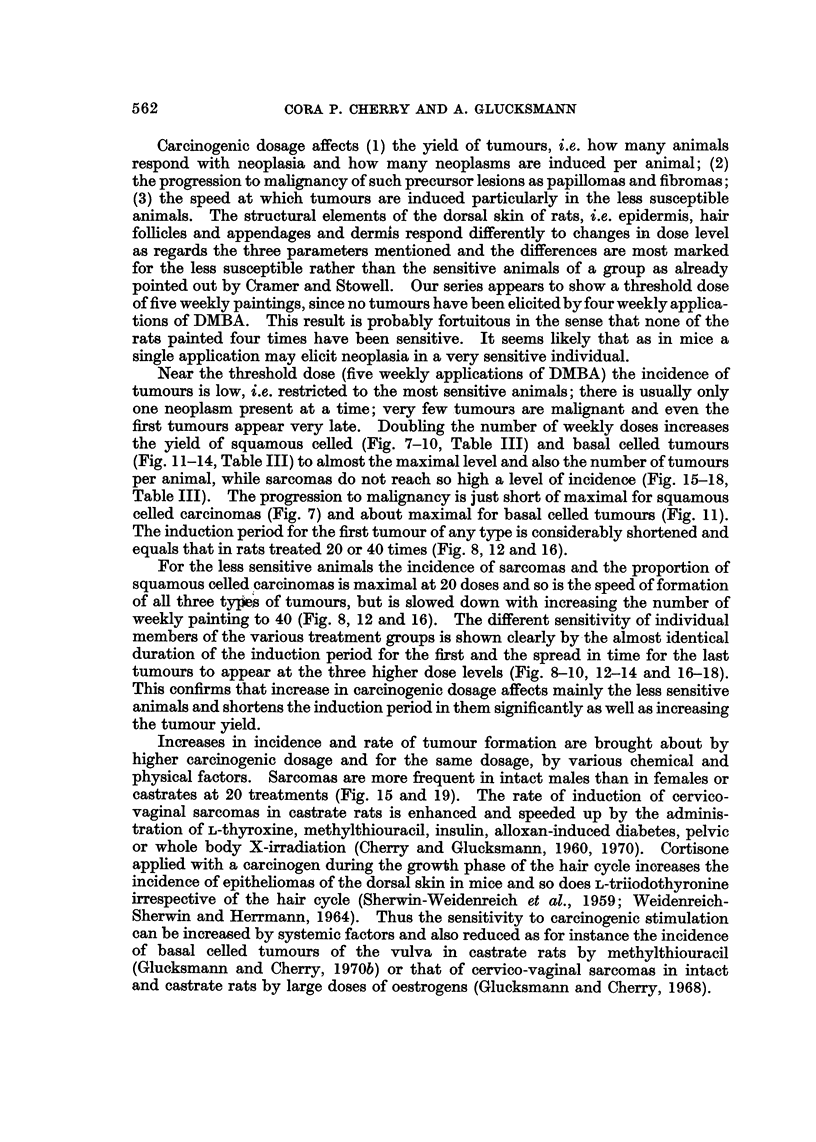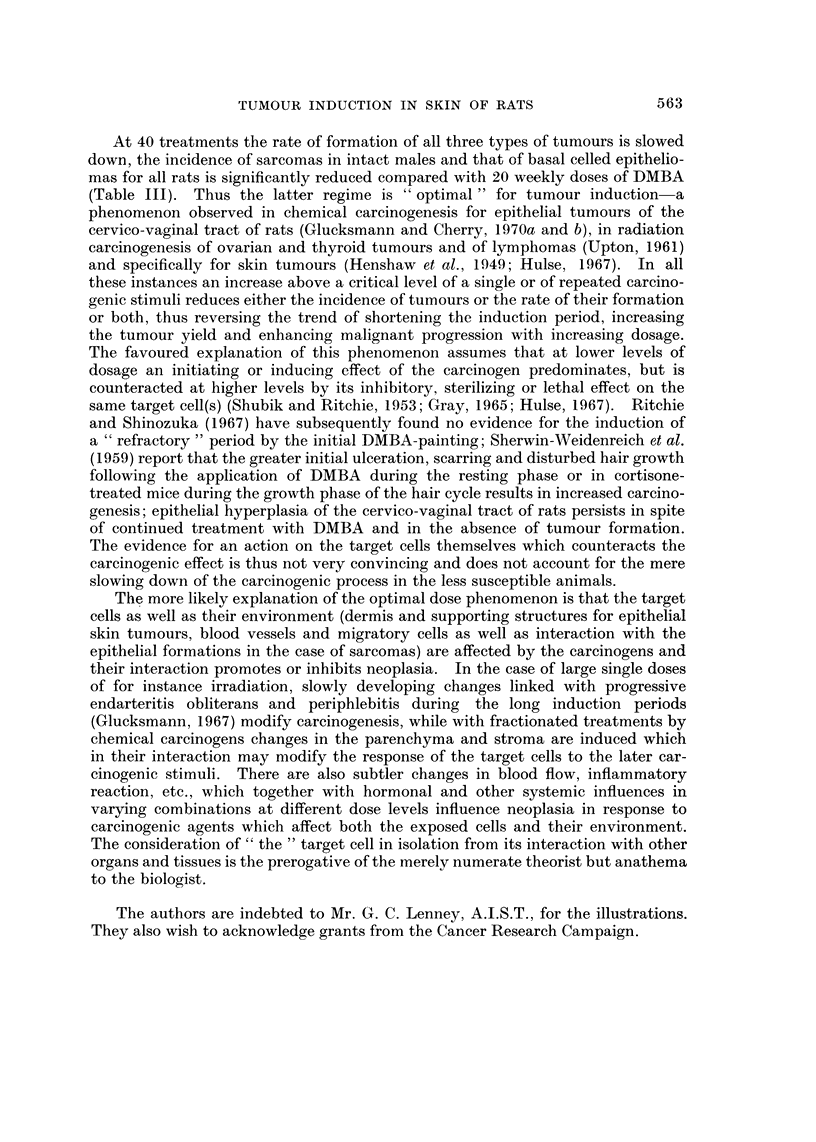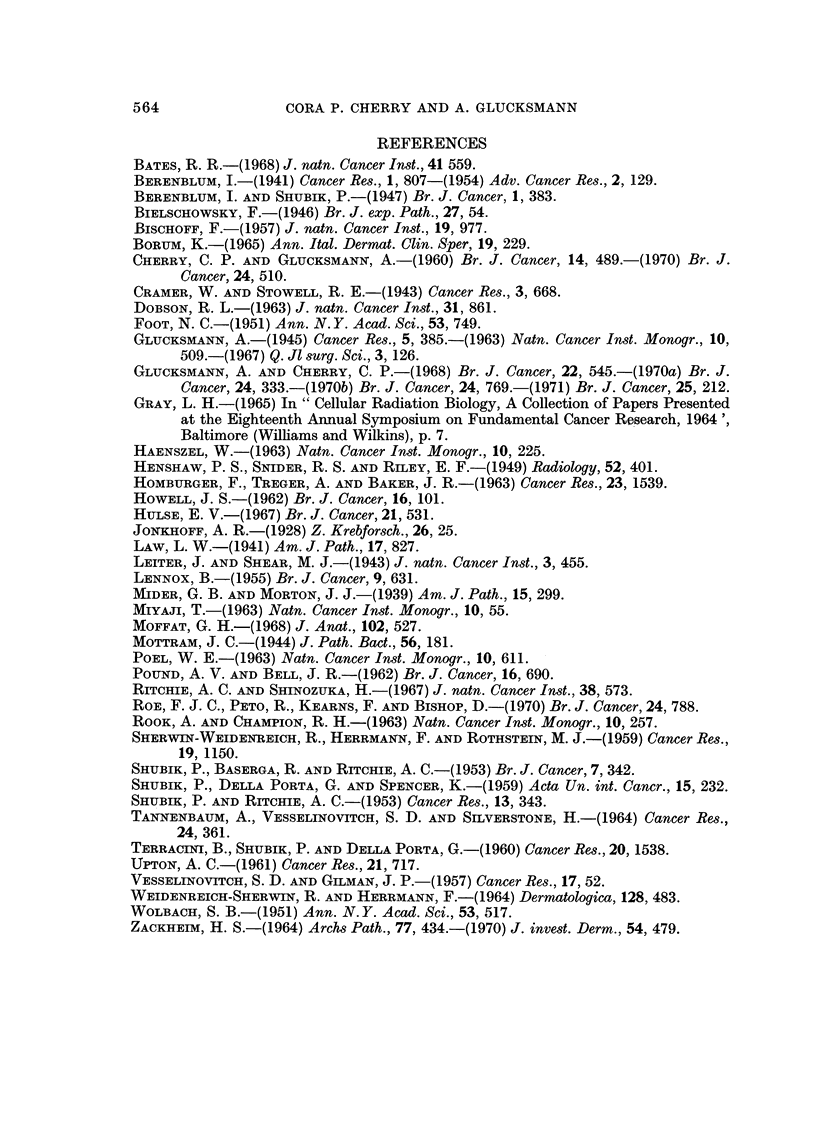# The Influence of Carcinogenic Dosage and of Sex on the Induction of Epitheliomas and Sarcomas in the Dorsal Skin of Rats

**DOI:** 10.1038/bjc.1971.70

**Published:** 1971-09

**Authors:** Cora P. Cherry, A. Glucksmann

## Abstract

**Images:**


### THE INFLUENCE OF CARCINOGENIC DOSAGE AND OF SEX ON THE INDUCTION OF EPITHELIOMAS AND SARCOMAS IN THE DORSAL SKIN OF RATS

CORA P. CHERRY AND A. GLUCKSMANN

*From the Strangeways Research Laboratory, Cambridge*

Received for publication May 15, 1971

**SUMMARY.—The effect of varying the numbers (4, 5, 10, 20 and 40) of weekly applications of DMBA to the dorsal skin of intact and castrate male and female rats on the induction of basal and squamous celled epitheliomas and of sarcomas has been investigated.**

**Basal celled tumours originate mainly in hair follicles and squamous celled neoplasms in the interfollicular regions of the epidermis and differ in their progression to malignancy. Penetration of the panniculus carnosus is neither a sufficient nor necessary criterion of malignancy since growing hair follicles pass through the muscle layer and carcinomas and sarcomas which are still confined to the dermis, spread along the perineural lymphatics and metastasise to the lungs.**

**Sex and castration do not affect carcinogenesis of epitheliomas in the dorsal skin at any dose level. Significantly more sarcomas result from 20 weekly paintings in male than in female or castrate rats.**

**The induction period for all tumour types is shortened in sensitive individuals only by an increase from 5 to 10 weekly applications. For less sensitive animals the rate of oncogenesis is accelerated with number of administrations up to 20, but slowed down from this level by 40 paintings. The optimal dose for speed of induction of all tumour types, for maximal yield of basal celled epitheliomas and for that of sarcomas in male rats is 20 weekly applications.**

**The progression to malignancy varies with tumour type: it is fast for sarcomas and slow for basal celled neoplasms. Of the 336 rats at risk only 1% have fibromas or other precursor lesions, while 40% have sarcomas; animals with squamous celled papillomas account for 12%, but those with carcinomas for 66%; there are, however, 64% of rats with basal celled papillomas and only 9% with carcinomas.**

**The optimal dose phenomenon in carcinogenesis is discussed.**

THE incidence of squamous celled carcinomas of the skin in men is about twice that in women (Haenszel, 1963; Miyaji, 1963). While the greater exposure of men to environmental factors favouring tumour development such as sunlight, occupational exposure to chemicals, injuries, may account for most of this difference, the endocrine-related biology of the skin in males and females may play a role in the sensitivity or responsiveness to external carcinogenic agents. Epithelial tumours in men (Rook and Champion, 1963; Foot, 1951) originate in hair follicles and since the replacement of lanugo by terminal hairs in various skin districts (face, chest, pubic region, legs, arms, etc.) is sex linked, it is not unlikely that sex hormones are involved in the prevalence of skin tumours in men. As spontaneous skin tumours are too rare in most laboratory animals, the influence of sex and the contribution of male characteristics to the enhanced risk of skin cancer has to be investigated on induced tumours. Since the hair coat of mice and rats does not reveal a sexual dimorphism comparable to that of man, only smaller sex differences may be expected in these species than in men.

Berenblum (1954) states that sex plays no significant role in skin carcinogenesis in most strains of mice, though in some strains males are somewhat more responsive than females. Most skin tumours in mice are papillomas and for the same carcinogenic treatment and dosage a greater incidence in males than females has been reported (Bates, 1968; Shubik, Della Porta and Spencer, 1959). Castration of males (Zackheim, 1970) reduces and that of females increases the yield of tumours (Bates, 1968). Induction of dermal sarcomas in male mice is obtained with lower doses of carcinogens than in females (Leiter and Shear, 1943), is faster in males (Homburger, Treger and Baker, 1963) and occurs in more males than females (Bischoff, 1957). These reports suggest an involvement of the male endocrine system in the greater response to carcinogenic stimulation. In other papers no sex difference or influence of castration has been found. This may be due to strain differences and to the use of carcinogenic doses in different solvents at or close to the " saturation " point which may obscure any sex difference.

Squamous cell carcinomas are induced in a small proportion of mice by carcinogenic chemicals $\pm$ croton oil: 3 of 570 tumours by Vesselinovitch and Gilman, 1957; 2 of 211 by Ritchie and Shinozuka, 1967; 100 of 899 by Shubik, Baserga and Ritchie, 1953; 30 to 50% by Cramer and Stowell, 1943; Terracini, Shubik and Della Porta, 1960; Glucksmann, 1963; Tannenbaum, Vesselinovitch and Silverstone, 1964. Exceptionally a figure of 100% has been reported by Poel (1963) using 19·0 to 470 $\mu$g. of benzopyrene in toluene three times a week for the life time of the animals. Fewer chemicals prove carcinogenic for the skin of rats than for that of mice, but with DMBA the incidence of carcinomas in rats is around 80% (Glucksmann, 1963; Zackheim, 1964) with weekly applications. While basal celled tumours are rare in mice, they occur quite frequently in rats (Bielschowsky, 1946; Lennox, 1955; Howell, 1962) and thus the tumour types of the rat skin resemble those of man more closely than do those in mice. At certain dose levels twice as many tumours are induced in rat salivary glands of males than in those of females and in males administration of oestrogens halves, while that of testosterone to females doubles the yield of neoplasms (Glucksmann and Cherry, 1971). The rat is thus a suitable animal for the testing of sex differences in tumour induction.

Experiments on dosage variations of chemical carcinogens have been carried out mostly in mice and have taken one of the following forms: (1) single applications of the carcinogen in various concentrations (Terracini *et al.*, 1960); (2) repeated applications of carcinogens at varying intervals (Cramer and Stowell, 1943) or (3) of various concentrations of the chemical over the life time of animals (Poel, 1963); (4) single (Mottram, 1944; Berenblum and Shubik, 1947) or (5) repeated applications of an " initiating " chemical carcinogen followed by repeated applications of croton oil as " promoting " agent for prolonged periods (30 to 50 weeks) (Berenblum, 1941; Shubik and Ritchie, 1953; Ritchie and Shinozuka, 1967; Vesselinovitch and Gilman, 1957). Even single paintings with methylcholanthrene (Mider and Morton, 1939) or DMBA (Law, 1941) induce tumours in very sensitive mice. The original claim that croton oil has no effect if applied before the carcinogen is not supported by recent experiments (Pound and Bell, 1962; Tannenbaum, Vesselinovitch and Silverstone, 1964) and according to

Shubik *et al.* (1959) " tumours induced in the special initiation-promotion studies are biologically very different from those seen in other studies employing only pure carcinogens ".

Apart from the single administration of various doses of carcinogens, the methods listed above are subject to the objection that treatment with either the carcinogens or the cocarcinogens is prolonged and carried out until visible neoplasms appear. Thus the " saturation point " for maximal tumour yield may be reached with doses far below the maximum and a great fraction of the carcinogenic stimulation may be wasted. Cramer and Stowell have pointed out that with the exception of minimal doses required for tumour induction in the most sensitive animals, an increase in carcinogenic dosage shortens the induction period in the more resistant animals and does not affect that in the sensitive mice; that with increasing dosage multiple neoplasms develop in extensive papillomatous regions, while with low doses single carcinomas arise in a skin showing only mild degrees of hyperplasia; that high susceptibility to carcinogenic stimuli may be restricted to only a small area of skin in an animal. If the period of observation is restricted to about 50 weeks the alternative objection may be made that tumours in the more resistant animals may not yet have appeared and that thus the total yield of tumours obtained, may not be a true measure of those induced.

Terracini *et al.* (1960) suggest that in mice the 30% incidence of carcinomas is maximal with a single dose of 200 $\mu$g. of DMBA in acetone and doses of 500 and 1000 $\mu$g. induce only 10 and 19% respectively. Similarly the maximal induction rate for sarcomas is 20% at 250 $\mu$g. and not changed by higher doses. This suggestion of an " optimal " dose phenomenon appears to be supported by a report of Shubik and Ritchie (1953) that in Swiss female mice painted once, twice or three times at weekly intervals with 0·2% DMBA in mineral oil followed by prolonged and repeated croton oil applications, the induction period increases with the number of initiating DMBA doses. The induction of a refractory state or a necrotizing effect of the DMBA treatment has been assumed as explanation. Subsequently Ritchie and Shinozuka (1967) and Vesselinovitch and Gilman (1957) have found that the yield of papillomas increases with number of DMBA paintings and that there is no evidence for the induction of a refractory period.

In our experiments we have explored the dosage phenomenon in carcinogenesis by restricting the number of weekly applications of DMBA to 5, 10 and 20 and contrasting them with weekly applications for the life span of the animals or until tumours appear, *i.e.* on the average with 40 applications as we have done in experiments on the female genital tract (Glucksmann and Cherry, 1970*a*, *b*). In this regime the interval between fractions and the concentration of DMBA are constant and the dose is varied by the number of fractions applied. This schedule allows of a better evaluation of the doses required to induce neoplasms in the various constituent tissues of the skin, to test for optimal dose levels and for sex differences at various doses. This report is concerned with the effect of varying doses of weekly DMBA applications on the induction of squamous and basal celled tumours and of sarcomas in the dorsal skin of intact and castrate male and female rats.

### MATERIALS AND METHODS

Intact and castrate male and female hooded rats of the Lister strain, random bred within a closed colony in this laboratory since 1940, were used for the experiments which extended over a period from 1953 to 1968. The rats were housed not more than seven to a cage and given water and food pellets of MRC-diet 86 *ad libitum*. Only animals surviving for at least 100 days after starting the experiment were considered at risk and the number of rats in the various treatment groups are given in Table I.

TABLE I.—*Controls and Treatment Groups with Sex, Number of Rats and Survival Data*

| Doses of DMBA | Sex | No. of rats | Survival in days: Range | Medium |
|---|---|---|---|---|
| None | ♂ | 38 | 321–906 | 654 |
| None | ♂̸ | 18 | 585–778 | 727 |
| None | ♀ | 36 | 322–826 | 719 |
| None | ♀̸ | 20 | 260–772 | 675 |
| 4 | ♂ | 18 | 302–803 | 558 |
| 4 | ♀ | 20 | 135–803 | 470 |
| 5 | ♂ | 21 | 350–729 | 634 |
| 5 | ♂̸ | 21 | 302–716 | 595 |
| 5 | ♀ | 21 | 344–722 | 540 |
| 5 | ♀̸ | 21 | 342–722 | 610 |
| 10 | ♂ | 21 | 246–604 | 396 |
| 10 | ♂̸ | 20 | 246–484 | 359 |
| 10 | ♀ | 21 | 210–604 | 356 |
| 10 | ♀̸ | 22 | 289–604 | 378 |
| 20 | ♂ | 34 | 163–330 | 261 |
| 20 | ♂̸ | 22 | 224–309 | 262 |
| 20 | ♀ | 20 | 179–329 | 260 |
| 20 | ♀̸ | 21 | 182–332 | 262 |
| 40 | ♂ | 30 | 188–313 | 261 |
| 40 | ♀ | 41 | 228–494 | 330 |

Castration of male and female rats was performed under ether anaesthesia at the age of 3 to 4 weeks. Carcinogenic treatment with a 1% solution in acetone of 9,10-dimethyl-1,2-benzanthracene (DMBA, Koch-Light Ltd or Sigma) was started when intact and castrate animals were aged 7 to 8 weeks. The carcinogenic solution was applied to the interscapular region of the dorsal skin without prior clipping or shaving of the hairs. In the early experiments the solution was distributed by means of a U-shaped pipette over the treated area; in later experiments the application was made by means of a cotton wool swab mounted on a thin wire rod, the swab was saturated with the solution which was distributed by a single stroke over the treated region of skin. This procedure was repeated at weekly intervals either for the life span of the animals (with an average of 40 applications) or restricted to 4, 5, 10 or 20 times (Table I). In two groups of intact males of which one was treated 20 times with the pipette and the other with the swab technique, the same incidence of tumours was obtained. This suggests that the dose of carcinogen at each application was similar for the two methods used.

All animals were examined at weekly intervals and those with clinical signs of malignant skin tumours, or if sick or aged, were killed and a post mortem performed. The entire treated area of skin with subcutaneous tissue was excised and stretched on pieces of filter paper before fixation in Zenker-acetic fluid. During the process of dehydration the skin was cut longitudinally into multiple narrow strips to include all tumours with a stretch of adjacent epidermis. In addition to the skin the following tissues were fixed for histological examination: pituitary, thyroid, thymus, liver, spleen, kidneys, adrenals, intestine, mesenteric nodes, gonads with uteri and cervico-vaginal tract or seminal vesicles and prostate. The material was fixed in Zenker-acetic or Bouin's fluid, processed in routine manner, embedded in paraffin and sectioned at 6 or 8 $\mu$ depending on organ; the endocrine glands were sectioned serially. Slides were stained with haematoxylin-eosin, the periodic acid-Schiff technique (PAS) after diastase digestion, Van Gieson or carmalum-orange G-aniline blue.

*Calculation of results*

With few weekly applications and thus low doses of the carcinogen generally only single tumours are induced and the rest of the treated skin is macroscopically and microscopically normal. With ten or more doses individual rats have macroscopic and microscopic papillomata as well as extensive papillomatous hyperplasia of the epidermis and multiple skin cancers which may have arisen by confluence of foci. It is thus difficult to assess accurately the number of tumours in individual animals. In the analysis the most advanced lesion was the criterion used in the classification of tumour-bearing rats. When animals had more than one distinct type of neoplasm they were recorded separately under sarcomas, squamous epitheliomas and basal celled tumours.

For the age-specific induction rates the percentage of tumour-bearing rats amongst those at risk for consecutive 100-day periods was plotted at the 50-day interval.

The survival data in Table I gives the age in days at death of the first and last animals in each group with median age; the duration of the experimental periods can be calculated by deducting 60 days from these figures.

*Incidence of skin tumours outside the painted area, of breast tumours and of leukaemia*

*Controls.*—Intact and castrate male and female rats of our colony have survived for periods ranging from 260 to 906 days (Table I). Spontaneous skin tumours do not occur in any of the four groups and breast tumours do not develop in the males or in the spayed females. In the intact females their incidence is 8% (Table II)

TABLE II.—*Incidence of Skin Tumours outside the Painted Area, of Breast Tumours and of Leukaemia*

| Doses of DMBA | Sex | No. of rats | Skin tumours No. | Skin tumours % | Breast tumours No. | Breast tumours % | Leukaemia No. | Leukaemia % |
|---|---|---|---|---|---|---|---|---|
| None | ♂ | 38 | 0 | 0 | 0 | 0 | 11 | 29 |
| None | ♂̸ | 18 | 0 | 0 | 0 | 0 | 2 | 11 |
| None | ♀ | 36 | 0 | 0 | 3 | 8 | 5 | 14 |
| None | ♀̸ | 20 | 0 | 0 | 0 | 0 | 5 | 25 |
| 4 | ♂ | 18 | 0 | 0 | 0 | 0 | 0 | 0 |
| 4 | ♀ | 20 | 1 | 5 | 2 | 10 | 0 | 0 |
| 5 | ♂ | 21 | 2 | 10 | 2 | 10 | 1 | 5 |
| 5 | ♂̸ | 21 | 2 | 10 | 0 | 0 | 2 | 10 |
| 5 | ♀ | 21 | 0 | 0 | 8 | 38 | 4 | 19 |
| 5 | ♀̸ | 21 | 1 | 5 | 0 | 0 | 2 | 10 |
| 10 | ♂ | 21 | 1 | 5 | 0 | 0 | 2 | 10 |
| 10 | ♂̸ | 20 | 0 | 0 | 0 | 0 | 1 | 5 |
| 10 | ♀ | 21 | 2 | 10 | 1 | 5 | 0 | 0 |
| 10 | ♀̸ | 22 | 0 | 0 | 0 | 0 | 8 | 36 |

and they have been found in rats surviving for at least 590 days. The type of leukaemia in controls has been described previously (Glucksmann and Cherry, 1968). The incidence varies from 11% to 29% and the disease is manifest clinically between 321 and 770 days of age.

*Skin tumours.*—Solitary tumours appeared on the skin outside the painted interscapular region in nine of the 374 experimental animals and only in those given ten or fewer applications of DMBA (Table II). Six rats had squamous celled carcinomas of which five arose on the face and one (♂ × 5 DMBA) on the hind leg. In three animals given five applications of DMBA benign neoplasms occurred on the ear; a squamous papilloma in a castrate male, a melanoma in an intact male and one in a spayed female.

In the groups treated four or five times with the carcinogen these epidermal tumours occured late, *i.e.* 574 to 657 days after the first painting. In the 3 rats given 10 doses the carcinomas were manifest clinically after 150, 248 and 325 days.

*Breast tumours* developed only in the groups given 4 to 10 weekly applications of DMBA to the dorsal skin in which the majority of animals survived for long periods (Tables I and II). While none occurred in the castrate females, two intact males had breast tumours, one an adenocarcinoma and the other a fibroma and they were found in rats aged 679 and 688 days. In two groups of intact females the incidence of these neoplasms is similar to that in the controls while in a third group it is much higher (38%). In other dose-response experiments for the induction of cervico-vaginal tumours and carried out at about the same time as the present series, the incidence of breast tumours in intacts given 5, 10 or 20 doses of DMBA was 40%, 33% and 19% respectively with corresponding median survival times of 561, 645 and 393 days. The lower incidence of these neoplasms with the highest dose may be accounted for by the shorter survival time of these animals.

*Leukaemia.*—The type and incidence of the disease is the same in controls and experimental animals and varies from 0% to 36% (Table II) with an age range of 210–626 days. In addition to the animals listed in the table, one intact female painted 20 times had leukaemia. There is no obvious influence of sex, castration and treatment with carcinogens on leukaemogenesis.

### RESULTS

### *Definition of malignancy*

For over 50 years the invasion or penetration of the panniculus carnosus has been and still is accepted as the main criterion of malignancy for epithelial skin tumours in rodents (Roe, Peto, Kearns and Bishop, 1970). It is assumed that normal epidermal structures and benign tumours are not found at this depth and that only few tumours with definite cytological and histological signs of malignancy are seen above the dermal muscle (Roe *et al.*, 1970). Neither of these assumptions is entirely correct.

In anagen hair follicles penetrate the panniculus carnosus (Fig. 1) or protrude through gaps in the muscle. Similarly benign papillomata may extend into and through the muscle layer. Squamous celled carcinomas arise in the hyperplastic epidermis, in abnormal hair follicles or most frequently in papillomatous lesions within the dermis. They do not change their biological, cytological or histological features on penetrating the panniculus carnosus and appear exactly the same above and below it. They invade the normal structures of the dermis as they do those of the subcutis and evidence of their malignancy is provided by the admittedly rare instances of metastasis to the lung of a squamous celled carcinoma still restricted to the dermis (Fig. 2 and 3). Invasion of the perineural lymphatics by carcinomas still confined to the dermis is a more frequent occurrence and also evidence of malignancy (Fig. 4, 5 and 6). Carcinomas within the dermis are not rare, if the animals are killed as soon as a definite clinical diagnosis of malignancy can be established, as is our practice. Thus 42% of 224 squamous celled carcinomas of the rat skin and 41% of those in mice are still restricted to the dermis. Penetration of the panniculus carnosus is obviously neither a sufficient nor a necessary criterion of malignancy for skin carcinomas in rodents.

Basal celled tumours of the dorsal skin like those of the vulva (Glucksmann and Cherry, 1970*b*) are derived mainly from hair follicles and like them in anagen, they grow as papillomas within an intact and usually thick basement membrane through the dermis (Moffat, 1968). They do not elicit an inflammatory reaction at this stage and merely push aside the normal dermal structures. Malignant basal celled epitheliomas are recognized by abnormalities and variability in cytological features, a high rate of mitosis, by the penetration of the basement membrane and the proliferation of individual cells and strands of them in the dermis and subcutis.

Hypertrophy, hyperplasia and adenomas of sebaceous glands are seen in almost all rats treated with ten or more doses of DMBA at weekly intervals. These tumours as well as three adenocarcinomas of sebaceous origin in our series have been omitted from the quantitative evaluation of our findings. Squamous celled tumours with admixtures of sebaceous origin and those containing a basal celled strain are recorded as squamous celled tumours. Trichoepitheliomas are counted as basal celled tumours since the latter too derive mainly from hair follicles in the rat.

Sarcomas arise either independently in the dermis or in the stroma of epidermal tumours which often shows a sarcomatous reaction. Independent growth away from the epithelial lesion, increased cellularity and rate of proliferation distinguish sarcomas from the sarcomatous changes in the stroma of epitheliomas. Most of the sarcomas are fibrosarcomas, though osteochondrosarcomas, myxofibrosarcomas,

---

EXPLANATION OF PLATES

FIG. 1.—Normal hair follicle in the rat skin penetrating the panniculus carnosus (p) by pushing between muscle fibres. PAS × 140.

FIG. 2.—Squamous celled carcinoma in the dorsal skin of a castrate male rat painted 10 times at weekly intervals with DMBA and killed 270 days after the first application. The tumour is confined to the dermis and does not touch the panniculus carnosus (p), but has metastasized to the lung. H. and E. × 27.

FIG. 3.—Lung metastasis from the squamous celled epidermal carcinoma illustrated in Fig. 2. H. and E. × 36.

FIG. 4.—Squamous celled carcinoma in the dorsal skin of a castrate male rat painted 10 times at weekly intervals with DMBA and killed 367 days after the first application. The tumour is still confined to the dermis and does not touch the panniculus carnosus (p). The diffuse spread of the carcinoma within the dermis (a) and in the perineural lymphatics (b) is illustrated in Fig. 5 and 6. H. and E. × 27.

FIG. 5.—Part of the tumour (a, in Fig. 4) at higher magnification. H. and E. × 140.

FIG. 6.—The perineural lymphatic (b in Fig. 4) is lined by tumour cells, one of which is in mitosis. H. and E. × 750.

Cherry and Glucksmann

Cherry and Glucksmann haemangioendotheliosarcomas and cellular types with giant cells occur occasionally. The sarcomas too tend to invade the perineural lymphatics and to spread along them.

*Squamous celled tumours*

The histogenesis of epidermal tumours proceeds via hyperplasia and radication of interfollicular regions to the formation of papillomas which project from the surface or intrude into the dermis or do both. Malignant changes occur in the papillomatous regions, usually in intruding papillomas or in merely hyperplastic skin regions. With ten and more weekly applications of DMBA the painted region is usually covered by multifocal papillomas which tend to become confluent. Malignant change is also very frequently multifocal and leads to the formation of apparently single carcinomas by confluence. It is impossible to assess with any degree of accuracy the number of papillomas in even serial sections of the skin, since they are so numerous and their borders are so ill-defined one from another. For this reason only animals with tumours are recorded rather than the number of tumours per rat which is at best only an approximation and certainly an understatement of their original number, *i.e.* before their confluence to a " unit " of macroscopic dimensions. In all individuals given ten or more paintings very numerous papillomas are present in addition to single or multiple carcinomas. With five weekly doses of DMBA there are usually only single papillomas or carcinomas in an epidermis which is only moderately hyperplastic. These findings

FIG. 7.—Percentage of squamous celled papillomas and carcinomas of the dorsal skin in intact (♂) and castrate (⚦) male and female (♀,⚦) rats induced by 5, 10, 20 or 40 weekly doses of DMBA.

are in full agreement with the statement by Cramer and Stowell (1943) that in mice smaller doses of carcinogens induce only single tumours in a fairly normal skin while larger doses elicit multiple tumours in a papillomatous epidermis.

The total incidence of animals with squamous celled papillomas or carcinomas is recorded in Fig. 7 for intact and castrate male and female rats given 5, 10, 20 or 40 weekly doses of DMBA. Male and female rats painted with DMBA four times at weekly intervals have failed to produce any skin tumours and any noticeable abnormality of the skin in over 2 years of observation. With five weekly doses a number of animals have reacted with tumours, some even with carcinomas. The incidence is, however, very much lower than in the groups given ten or more applications which have not only more tumour bearing animals, but also more tumours per animal and a much higher proportion of carcinomas to papillomas,

Fig. 8.—Cumulative incidence of squamous celled neoplasms (papillomas + carcinomas) in male (♂) and female (♀) rats induced by 5, 10, 20 or 40 weekly applications of DMBA.

*i.e.* in a higher proportion of rats the skin has advanced to the malignant state in the process of carcinogenesis. There are no significant differences in these three treatment groups as to incidence of squamous celled tumours, proportion of papillomas to carcinomas or with sex or castration of the animals (Fig. 7). It is noteworthy that in the great majority of rats painted ten or more times carcinogenesis progresses to full malignancy while in mice given a single painting of DMBA followed by frequently repeated paintings with croton oil only about 11% of the induced skin tumours become malignant (Shubik, Baserga and Ritchie, 1953).

The rate of cumulative increase in incidence of squamous celled tumours of which the majority are carcinomas is plotted for intact male and female rats in

Fig. 8. The incidence is almost identical and since the castrate male and female animals behave identically as regards cumulative incidence of tumours, the curves for them are omitted. In all four groups the rate of tumour induction increases with number of DMBA doses up to the 20 dose level, but actually slows down subsequently with 40 doses in intact animals. The optimal dose phenomenon in tumour induction is also revealed if the age-specific tumour incidence is plotted for intact males (Fig. 9) or intact females (Fig. 10). Since the experiment with 40 doses of DMBA given to females has been repeated at an interval of 5 years with identical results and that with 20 doses to males at an interval of 4 years, the findings of an optimal dose for skin tumour induction can be regarded as well established.

Fig. 9.—Age-specific induction rates of squamous celled tumours in intact male rats for 5, 10, 20 or 40 weekly doses of DMBA.

Increasing the carcinogenic dosage shortens the induction period only for the less sensitive animals (Cramer and Stowell, 1943) but does not affect that of the most sensitive individuals. This phenomenon is clearly seen in Fig. 8–10, where the spread for the appearance of the first tumours in rats painted ten or more times is much smaller than that for the last tumours to appear, *i.e.* in the least sensitive individuals. With five paintings only the most sensitive rats develop tumours and these appear much later than with the greater number of DMBA administrations in all four groups of animals. The differences in the incidence of squamous celled tumours between males and females and intacts and castrates are probably due to the chance distribution in the groups of particularly sensitive rats (Fig. 7). Such a chance difference in the presence of sensitive animals may also account for

FIG. 10.—Age-specific induction rates of squamous celled neoplasms in intact female rats painted 5, 10, 20 or 40 times with DMBA at weekly intervals.

the fact that no tumours are obtained with four weekly doses and some 30% with five weekly doses. In mice a single application of a carcinogenic hydrocarbon may elicit tumours in sensitive animals and it seems probable that such a result may be obtained also with very sensitive rats.

*Basal celled tumours*

The majority of papilloma arise in isolated and usually not adjacent hair follicles and grow within their basement membrane and adjacent connective tissue sheath. Individual foci are separated by the relatively large interfollicular areas of skin and because of their separation and manner of expansion have less tendency to become confluent than squamous celled papillomas or foci. Nevertheless multiple, but widely spaced basal celled papillomas are found in most rats. Though they could be enumerated, we have adhered to the evaluation of number of rats with tumours as for squamous neoplasms rather than to give an estimate of number of basal celled tumours per rat. Relatively few of the neoplasms become malignant (Fig. 11) when they burst through their basement membranes and connective tissue sheath and tend to become confluent.

The total incidence of tumours increases with number of paintings up to and including the $\times$ 20 level and subsequently drops significantly by 20% $\pm$ 9·3 in males and 16% $\pm$ 7·9 in females. Malignant conversion is low with five doses but rises to about the same level with more numerous applications of DMBA. The rate of tumour development accelerates with increasing number of doses up to 20, but slows down with 40 paintings (Fig. 12). The same optimal rate is

FIG. 11.—Percentage of basal celled papillomas and carcinomas of the dorsal skin induced in intact (♂) and castrate (♂̸) males and females (♀, ♀̸) by 5, 10, 20 or 40 weekly doses of DMBA.

FIG. 12.—Cumulative incidence of basal celled neoplasms (papillomas and carcinomas) in male and female rats painted 5, 10, 20 or 40 times with DMBA at weekly intervals.

seen in the age-specific plots of carcinogenesis for males (Fig. 13) and for females (Fig. 14). There is very little difference in rate of tumour development for 10 and 40 doses of DMBA, while 20 doses accelerate and increase tumour formation above this level. There is no difference with sex or castration for the same number of doses either as regards speed or incidence of basal celled neoplasms, nor is there any consistent variation in the proportion of carcinomas to papillomas with sex (Fig. 11). A similar optimal dose of 20 paintings for total incidence and rate of induction of basal celled tumours has been found in the vulva of rats (Glucksmann and Cherry, 1970*b*) and this pattern has not been affected by treatment with thyroactive compounds. At this site, however, the proportion of carcinomas to papillomas is significantly greater with 20 than with 40 doses.

FIG. 13.—Age-specific induction rates of basal celled tumours in intact males for 5, 10, 20 or 40 weekly doses of DMBA.

*Sarcomas*

There is usually only a single sarcoma per rat though this fact does not necessarily imply origin in a single focus or cell. As at other sites such as the cervico-vaginal tract (Glucksmann and Cherry, 1970*a*) or the salivary glands (Glucksmann and Cherry, 1971) the histological type of the tumour varies from a well differentiated fibrosarcoma to an anaplastic cellular tumour and may also entail a component of osteochondrosarcoma, myxofibrosarcoma or haemangio-endotheliosarcoma. The presence of multiple components in the same neoplasm may be due to a multicellular or multifocal origin, *i.e.* the carcinogenic treatment affects a variety of tissues or cells, or alternatively to a pluripotentiality of affected sarcomatous cells which subsequently diverge in their differentiation or attempt at differentiation according to the environment. There is at the moment not

FIG. 14.—Age-specific induction rates of basal celled tumours in intact females for 5, 10, 20 or 40 weekly doses of DMBA.

FIG. 15.—Percentage of dermal sarcomas of the dorsal skin induced in intact and castrate males and females by 5, 10, 20 or 40 weekly applications of DMBA.

sufficient evidence to distinguish between these possibilities. In the salivary glands particularly, and to a great extent also in the cervico-vaginal tract, the variation in type of induced sarcomas is much greater than in the dorsal skin, where the majority of tumours (*i.e.* 126 of 133) are fibrosarcomas of varying degree of maturation. The remaining 7 tumours are: 5 osteochondrofibrosarcomas, 1 myxofibrosarcoma and 1 haemangioendotheliosarcoma. There is no clear correlation of tumour type with DMBA dosage or with sex of the rats.

The histogenesis of the dermal sarcomas like that of the cervico-vaginal tract progresses only very rarely via the stage of a fibromatous precursor lesion. In the 336 rats at risk fibromas are present in only three animals (1%) as against 133 with sarcomas (40%) and there is also one case of haemangioma. Many sarcomas arise in the stroma of squamous celled neoplasms, but others originate independently of epitheliomas in the dermis. The precursor lesions in the form of cellularity of the tumour stroma are fairly rare, presumably because unlike the epithelial tumours the connective tissue neoplasms progress rapidly to the malignant state. Because of the scarcity of fibromas and precursor lesions they have been omitted from the quantitative evaluation.

The incidence of sarcomas increases up to 20 weekly doses of DMBA and then falls significantly in the case of intact males and less so in intact females (Fig. 15). The increase from 5 to 10 and 20 applications is significant if all rats thus treated are considered (Table III). While sex differences are not significant at 5, 10 and 40 paintings, at 20 doses significantly more males than females have sarcomas and the castrate rats have fewer than their respective intact sex (Fig. 15). The rate of tumour formation is optimal at × 20 DMBA and, as with the epithelial neoplasms, that at 40 doses is about the same as for ten (Fig. 16). A suggestion of sex difference for intact males and females is seen in the age-specific plot of sarcomas: at

FIG. 16.—Cumulative incidence of dermal sarcomas in male and female rats painted 5, 10, 20 or 40 times with DMBA at weekly intervals.

FIG. 17.—Age-specific induction rates of dermal sarcomas in intact males for 5, 10, 20 or 40 weekly doses of DMBA.

FIG. 18.—Age-specific induction rates of dermal sarcomas in intact females for 5, 10, 20 or 40 weekly doses of DMBA.

TABLE III.—*Incidence of Squamous Celled Tumours and of Sarcomas for Different Doses of DMBA*

| DMBA | Sex | No. at risk | Squamous celled tumours % | Sarcomas % | Basal celled tumours % |
|---|---|---|---|---|---|
| ×40 | ♂, ♀ | 71 | 96±2·3 | 55±5·9 | 73±5·3 |
| ×20 | ♂, ♂̸, ♀, ♀ | 97 | 99±1·0 | 66±4·8 | 92±2·8 |
| ×10 | ♂, ♂̸, ♀, ♀̸ | 84 | 96±2·1 | 27±4·8 | 85±3·9 |
| ×5 | ♂, ♂̸, ♀, ♀̸ | 84 | 24±4·7* | 8±3·0 | 31±5·1 |

* Carcinomas only: 5 ± 2·4.

all doses of DMBA there is a rise in percentage to about 80% in males and to only about 40% in females (Fig. 17 and 18). The cumulative percentage shows this difference only at 20 weekly doses of DMBA (Fig. 16), and the same is true for the dose-response curve (Fig. 19). For the same incidence of squamous celled carcinomas in intact males and females, the percentage of induced sarcomas is only about half that of epitheliomas except for intact males painted 20 times.

The relative proportion of carcinomas to sarcomas varies by a factor of 1·5 to 3·5 for different treatment regimes and of 2 (265 squamous celled papillomas plus carcinomas to 133 sarcomas) for the whole series. Most of the epitheliomas are malignant (Fig. 7) except for the lowest dose (Table III) when an incidence of 5% carcinomas is not really different from that of 8% sarcomas. The total percentage of carcinomas is 66% (224 rats with squamous celled carcinomas) as against 40% for sarcomas (133 rats with sarcomas), *i.e.* a factor of about 1·7.

### DISCUSSION

In rats sex has a significant influence on the induction of sarcomas in the dorsal skin by 20 paintings with DMBA. Tumour formation occurs more frequently in males than in females or castrates (Fig. 16–19) and this sex difference parallels that in man (Haenszel, 1963; Miyaji, 1963; Rook and Champion, 1963) and mice (Bates, 1968; Shubik *et al.*, 1959; Zackheim, 1970; Leiter and Shear, 1943; Homburger *et al.*, 1963; Bischoff, 1957). As expected from the absence of a dimorphism in the hair coat of rats, no sex difference in the induction of epithelial tumours comparable to that in man is seen at least in the dorsal region and with the dose regime employed. In the vulval skin of rats castration enhances the progression to malignancy of squamous celled tumours elicited by five and ten weekly paintings with DMBA (Glucksmann and Cherry, 1970*a*), but consistently reduces the incidence of basal celled neoplasms at all dose levels of DMBA. With 40 paintings, for instance, there are 44 basal celled epitheliomas in 64 intacts (69%), but only 9 in 36 castrates (25%). The relation of these tumours to hair follicles will be discussed later. These observations indicate that sex hormones may affect carcinogenesis in the skin, that they are effective under varying conditions of stimulation and vary with the component tissues and regions of the skin. They represent only one of the many interacting factors involved in the induction of neoplasia.

The proportions of benign to malignant lesions in the rat skin vary greatly with the tissue of origin of the tumours; benign precursor lesions and fibromas are rare (about 1%) compared with sarcomas (about 40%) suggesting a rapid progression to malignancy. At all dose levels basal celled papillomas predominate, *i.e.* in 214 or 64% of 336 rats at risk, while carcinomas are relatively rare, *i.e.* in 30 of

336 animals or 9%. In the same group of rats 224 have squamous celled carcinomas (66%) and 41 papillomas (12%). These figures are based on the presence of the most advanced lesion in an individual and those with carcinomas usually have papillomas and those with sarcomas may have occasionally less advanced lesions as well. The total incidence of tumours in these rats increases from 40% with sarcomas, to 73% with basal celled and 78% with squamous celled epitheliomas. In rats the squamous celled tumours arise mainly from the interfollicular areas of the epidermis which are larger than in mice because of the differences in the density of the pelage: rats have fewer but coarser hairs per unit area, while mice have more and finer hairs, and thus relatively less interfollicular epidermis. In mice hair follicles and interfollicular regions participate in the formation of squamous papillomas and carcinomas and the growth rate of papillomas varies with the hair cycle (Jonkhoff, 1928; Glucksmann, 1945; Wolbach, 1951; Borum, 1965). In rats hair follicles and the interfollicular regions of the epidermis are not as closely interwoven as in mice, react more independently of one another and give rise to different tumour types. Basal celled tumours in mice are rare (Lennox, 1955; Bielschowsky, 1946) and in our series amount to merely 4%; in rats they arise in hair follicles (Howell, 1962; Dobson, 1963) preferentially. It is interesting to speculate whether the relatively lower rate of progression to malignancy of squamous celled tumours in mice and of basal celled epitheliomas in rats is linked to their origin in hair follicles. It is not known whether carcinogenesis or rate of growth of rat tumours is affected by the hair cycle as it is in mice.

FIG. 19.—Dose-response curve for the induction of squamous celled carcinomas and of dermal sarcomas in intact male and female rats.

Carcinogenic dosage affects (1) the yield of tumours, *i.e.* how many animals respond with neoplasia and how many neoplasms are induced per animal; (2) the progression to malignancy of such precursor lesions as papillomas and fibromas; (3) the speed at which tumours are induced particularly in the less susceptible animals. The structural elements of the dorsal skin of rats, *i.e.* epidermis, hair follicles and appendages and dermis respond differently to changes in dose level as regards the three parameters mentioned and the differences are most marked for the less susceptible rather than the sensitive animals of a group as already pointed out by Cramer and Stowell. Our series appears to show a threshold dose of five weekly paintings, since no tumours have been elicited by four weekly applications of DMBA. This result is probably fortuitous in the sense that none of the rats painted four times have been sensitive. It seems likely that as in mice a single application may elicit neoplasia in a very sensitive individual.

Near the threshold dose (five weekly applications of DMBA) the incidence of tumours is low, *i.e.* restricted to the most sensitive animals; there is usually only one neoplasm present at a time; very few tumours are malignant and even the first tumours appear very late. Doubling the number of weekly doses increases the yield of squamous celled (Fig. 7–10, Table III) and basal celled tumours (Fig. 11–14, Table III) to almost the maximal level and also the number of tumours per animal, while sarcomas do not reach so high a level of incidence (Fig. 15–18, Table III). The progression to malignancy is just short of maximal for squamous celled carcinomas (Fig. 7) and about maximal for basal celled tumours (Fig. 11). The induction period for the first tumour of any type is considerably shortened and equals that in rats treated 20 or 40 times (Fig. 8, 12 and 16).

For the less sensitive animals the incidence of sarcomas and the proportion of squamous celled carcinomas is maximal at 20 doses and so is the speed of formation of all three types of tumours, but is slowed down with increasing the number of weekly painting to 40 (Fig. 8, 12 and 16). The different sensitivity of individual members of the various treatment groups is shown clearly by the almost identical duration of the induction period for the first and the spread in time for the last tumours to appear at the three higher dose levels (Fig. 8–10, 12–14 and 16–18). This confirms that increase in carcinogenic dosage affects mainly the less sensitive animals and shortens the induction period in them significantly as well as increasing the tumour yield.

Increases in incidence and rate of tumour formation are brought about by higher carcinogenic dosage and for the same dosage, by various chemical and physical factors. Sarcomas are more frequent in intact males than in females or castrates at 20 treatments (Fig. 15 and 19). The rate of induction of cervico-vaginal sarcomas in castrate rats is enhanced and speeded up by the administration of L-thyroxine, methylthiouracil, insulin, alloxan-induced diabetes, pelvic or whole body X-irradiation (Cherry and Glucksmann, 1960, 1970). Cortisone applied with a carcinogen during the growth phase of the hair cycle increases the incidence of epitheliomas of the dorsal skin in mice and so does L-triiodothyronine irrespective of the hair cycle (Sherwin-Weidenreich *et al.*, 1959; Weidenreich-Sherwin and Herrmann, 1964). Thus the sensitivity to carcinogenic stimulation can be increased by systemic factors and also reduced as for instance the incidence of basal celled tumours of the vulva in castrate rats by methylthiouracil (Glucksmann and Cherry, 1970*b*) or that of cervico-vaginal sarcomas in intact and castrate rats by large doses of oestrogens (Glucksmann and Cherry, 1968).

At 40 treatments the rate of formation of all three types of tumours is slowed down, the incidence of sarcomas in intact males and that of basal celled epitheliomas for all rats is significantly reduced compared with 20 weekly doses of DMBA (Table III). Thus the latter regime is " optimal " for tumour induction—a phenomenon observed in chemical carcinogenesis for epithelial tumours of the cervico-vaginal tract of rats (Glucksmann and Cherry, 1970*a* and *b*), in radiation carcinogenesis of ovarian and thyroid tumours and of lymphomas (Upton, 1961) and specifically for skin tumours (Henshaw *et al.*, 1949; Hulse, 1967). In all these instances an increase above a critical level of a single or of repeated carcinogenic stimuli reduces either the incidence of tumours or the rate of their formation or both, thus reversing the trend of shortening the induction period, increasing the tumour yield and enhancing malignant progression with increasing dosage. The favoured explanation of this phenomenon assumes that at lower levels of dosage an initiating or inducing effect of the carcinogen predominates, but is counteracted at higher levels by its inhibitory, sterilizing or lethal effect on the same target cell(s) (Shubik and Ritchie, 1953; Gray, 1965; Hulse, 1967). Ritchie and Shinozuka (1967) have subsequently found no evidence for the induction of a " refractory " period by the initial DMBA-painting; Sherwin-Weidenreich *et al.* (1959) report that the greater initial ulceration, scarring and disturbed hair growth following the application of DMBA during the resting phase or in cortisone-treated mice during the growth phase of the hair cycle results in increased carcinogenesis; epithelial hyperplasia of the cervico-vaginal tract of rats persists in spite of continued treatment with DMBA and in the absence of tumour formation. The evidence for an action on the target cells themselves which counteracts the carcinogenic effect is thus not very convincing and does not account for the mere slowing down of the carcinogenic process in the less susceptible animals.

The more likely explanation of the optimal dose phenomenon is that the target cells as well as their environment (dermis and supporting structures for epithelial skin tumours, blood vessels and migratory cells as well as interaction with the epithelial formations in the case of sarcomas) are affected by the carcinogens and their interaction promotes or inhibits neoplasia. In the case of large single doses of for instance irradiation, slowly developing changes linked with progressive endarteritis obliterans and periphlebitis during the long induction periods (Glucksmann, 1967) modify carcinogenesis, while with fractionated treatments by chemical carcinogens changes in the parenchyma and stroma are induced which in their interaction may modify the response of the target cells to the later carcinogenic stimuli. There are also subtler changes in blood flow, inflammatory reaction, etc., which together with hormonal and other systemic influences in varying combinations at different dose levels influence neoplasia in response to carcinogenic agents which affect both the exposed cells and their environment. The consideration of " the " target cell in isolation from its interaction with other organs and tissues is the prerogative of the merely numerate theorist but anathema to the biologist.

The authors are indebted to Mr. G. C. Lenney, A.I.S.T., for the illustrations. They also wish to acknowledge grants from the Cancer Research Campaign.

### REFERENCES

BATES, R. R.—(1968) *J. natn. Cancer Inst.*, **41** 559.
BERENBLUM, I.—(1941) *Cancer Res.*, **1**, 807—(1954) *Adv. Cancer Res.*, **2**, 129.
BERENBLUM, I. AND SHUBIK, P.—(1947) *Br. J. Cancer*, **1**, 383.
BIELSCHOWSKY, F.—(1946) *Br. J. exp. Path.*, **27**, 54.
BISCHOFF, F.—(1957) *J. natn. Cancer Inst.*, **19**, 977.
BORUM, K.—(1965) *Ann. Ital. Dermat. Clin. Sper*, **19**, 229.
CHERRY, C. P. AND GLUCKSMANN, A.—(1960) *Br. J. Cancer*, **14**, 489.—(1970) *Br. J. Cancer*, **24**, 510.
CRAMER, W. AND STOWELL, R. E.—(1943) *Cancer Res.*, **3**, 668.
DOBSON, R. L.—(1963) *J. natn. Cancer Inst.*, **31**, 861.
FOOT, N. C.—(1951) *Ann. N.Y. Acad. Sci.*, **53**, 749.
GLUCKSMANN, A.—(1945) *Cancer Res.*, **5**, 385.—(1963) *Natn. Cancer Inst. Monogr.*, **10**, 509.—(1967) *Q. Jl surg. Sci.*, **3**, 126.
GLUCKSMANN, A. AND CHERRY, C. P.—(1968) *Br. J. Cancer*, **22**, 545.—(1970*a*) *Br. J. Cancer*, **24**, 333.—(1970*b*) *Br. J. Cancer*, **24**, 769.—(1971) *Br. J. Cancer*, **25**, 212.
GRAY, L. H.—(1965) In " Cellular Radiation Biology, A Collection of Papers Presented at the Eighteenth Annual Symposium on Fundamental Cancer Research, 1964 ', Baltimore (Williams and Wilkins), p. 7.
HAENSZEL, W.—(1963) *Natn. Cancer Inst. Monogr.*, **10**, 225.
HENSHAW, P. S., SNIDER, R. S. AND RILEY, E. F.—(1949) *Radiology*, **52**, 401.
HOMBURGER, F., TREGER, A. AND BAKER, J. R.—(1963) *Cancer Res.*, **23**, 1539.
HOWELL, J. S.—(1962) *Br. J. Cancer*, **16**, 101.
HULSE, E. V.—(1967) *Br. J. Cancer*, **21**, 531.
JONKHOFF, A. R.—(1928) *Z. Krebforsch.*, **26**, 25.
LAW, L. W.—(1941) *Am. J. Path.*, **17**, 827.
LEITER, J. AND SHEAR, M. J.—(1943) *J. natn. Cancer Inst.*, **3**, 455.
LENNOX, B.—(1955) *Br. J. Cancer*, **9**, 631.
MIDER, G. B. AND MORTON, J. J.—(1939) *Am. J. Path.*, **15**, 299.
MIYAJI, T.—(1963) *Natn. Cancer Inst. Monogr.*, **10**, 55.
MOFFAT, G. H.—(1968) *J. Anat.*, **102**, 527.
MOTTRAM, J. C.—(1944) *J. Path. Bact.*, **56**, 181.
POEL, W. E.—(1963) *Natn. Cancer Inst. Monogr.*, **10**, 611.
POUND, A. V. AND BELL, J. R.—(1962) *Br. J. Cancer*, **16**, 690.
RITCHIE, A. C. AND SHINOZUKA, H.—(1967) *J. natn. Cancer Inst.*, **38**, 573.
ROE, F. J. C., PETO, R., KEARNS, F. AND BISHOP, D.—(1970) *Br. J. Cancer*, **24**, 788.
ROOK, A. AND CHAMPION, R. H.—(1963) *Natn. Cancer Inst. Monogr.*, **10**, 257.
SHERWIN-WEIDENREICH, R., HERRMANN, F. AND ROTHSTEIN, M. J.—(1959) *Cancer Res.*, **19**, 1150.
SHUBIK, P., BASERGA, R. AND RITCHIE, A. C.—(1953) *Br. J. Cancer*, **7**, 342.
SHUBIK, P., DELLA PORTA, G. AND SPENCER, K.—(1959) *Acta Un. int. Cancr.*, **15**, 232.
SHUBIK, P. AND RITCHIE, A. C.—(1953) *Cancer Res.*, **13**, 343.
TANNENBAUM, A., VESSELINOVITCH, S. D. AND SILVERSTONE, H.—(1964) *Cancer Res.*, **24**, 361.
TERRACINI, B., SHUBIK, P. AND DELLA PORTA, G.—(1960) *Cancer Res.*, **20**, 1538.
UPTON, A. C.—(1961) *Cancer Res.*, **21**, 717.
VESSELINOVITCH, S. D. AND GILMAN, J. P.—(1957) *Cancer Res.*, **17**, 52.
WEIDENREICH-SHERWIN, R. AND HERRMANN, F.—(1964) *Dermatologica*, **128**, 483.
WOLBACH, S. B.—(1951) *Ann. N.Y. Acad. Sci.*, **53**, 517.
ZACKHEIM, H. S.—(1964) *Archs Path.*, **77**, 434.—(1970) *J. invest. Derm.*, **54**, 479.